# Evolutionary, genetic, structural characterization and its functional implications for the influenza A (H1N1) infection outbreak in India from 2009 to 2017

**DOI:** 10.1038/s41598-019-51097-w

**Published:** 2019-10-11

**Authors:** Sara Jones, Shijulal Nelson-Sathi, Yejun Wang, Raji Prasad, Sabrina Rayen, Vibhuti Nandel, Yueming Hu, Wei Zhang, Radhakrishnan Nair, Sanjai Dharmaseelan, Dhanya Valaveetil Chirundodh, Rakesh Kumar, Radhakrishna Madhavan Pillai

**Affiliations:** 1 Pathogen Biology Program, Rajiv Gandhi Center for Biotechnology, Thiruvananthapuram, Kerala 695014 India; 2 Interdiciplinary Biology Program, Rajiv Gandhi Center for Biotechnology, Thiruvananthapuram, Kerala 695014 India; 3 Department of Cell Biology and Genetics, School of Basic Medical Sciences, Shenzhen University Health Science Center, Shenzhen, 518060 China; 4 Shenzhen Gen Read Technology, Shenzhen, 518000 China; 5 Laboratory Medicine and Molecular Diagnostics Program, Rajiv Gandhi Centre for Biotechnology, Thiruvananthapuram, Kerala 695014 India; 6 Cancer Research Program, Rajiv Gandhi Centre for Biotechnology, Thiruvananthapuram, Kerala 695014 India

**Keywords:** Microbiology, Influenza virus

## Abstract

Influenza A (H1N1) continues to be a major public health threat due to possible emergence of a more virulent H1N1 strain resulting from dynamic changes in virus adaptability consequent to functional mutations and antigenic drift in the hemagglutinin (HA) and neuraminidase (NA) surface proteins. In this study, we describe the genetic and evolutionary characteristics of H1N1 strains that circulated in India over a period of nine years from 2009 to 2017 in relation to global strains. The finding is important from a global perspective since previous phylogenetic studies have suggested that the tropics contributed substantially to the global circulation of influenza viruses. Bayesian phylogenic analysis of HA sequences along with global strains indicated that there is a temporal pattern of H1N1 evolution and clustering of Indian isolates with globally circulating strains. Interestingly, we observed four new amino acid substitutions (S179N, I233T, S181T and I312V) in the HA sequence of H1N1 strains isolated during 2017 and two (S181T and I312V) were found to be unique in Indian isolates. Structurally these two unique mutations could lead to altered glycan specificity of the HA gene. Similarly, sequence and structural analysis of NA domain revealed that the presence of K432E mutation in H1N1 strains isolated after 2015 from India and in global strains found to induce a major loop shift in the vicinity of the catalytic site. The findings presented here offer an insight as to how these acquired mutations could be associated to an improved adaptability of the virus for efficient human transmissibility.

## Introduction

Among viral respiratory illnesses, influenza A (H1N1) - a single stranded RNA virus - continues to be a major public health threat worldwide^1^. Annually, influenza virus infects about three to five million individuals globally, leading to approximately 650,000 deaths^2,3^. The first influenza pandemic of the 21^st^ century broke in Mexico in 2009, as a result of re-assortment between avian, human, and swine influenza viruses to develop a new strain of Influenza A (H1N1)^4^. Within a few weeks, the infection spread throughout the world, affecting nearly 214 countries and affecting the population with little or no immunity^5^. While Influenza A (H1N1) was not lethal per say, its ability to spread worldwide in a short period highlighted the public health threat posed by this virus. The temperate regions reported higher incidences of the human influenza viral infection during the winter season (December – March), while tropical and subtropical areas displayed a year round circulation and biannual peaks^6,7^. Reports from Kerala the southern state of India, often shows two distinct peaks of influenza infections during the north-east monsoon months of September – November and during the summer months of May – July^8^. During early 2014, India saw a mild influenza season, followed by a huge flare up in 2015^9–11^, followed by a spike in 2017 during the winter months of December – March^12–14^. This raised speculation about potential mutations in the hemagglutinin (HA) and neuraminidase (NA) surface glycoproteins on the virus which act as the primary immunity-eliciting antigens that are responsible for the inflection^15,16^.

The HA and NA proteins undergo selection pressure, mutagenesis, antigenic drift, and adaptation with each round of outbreaks^17–20^. The HA1 (globular in shape) and HA2 (fibrous stem-like structure) domains participate in anchoring the HA protein into the viral lipid envelope^21^ and are crucial for initiating virus-host interactions. In contrast, NA is a homo-tetramer of 470 amino acids that form the cytoplasmic, transmembrane, head and stem domains of the virus with a role in preventing the aggregation of viral particles at the time of budding of progeny viruses^22^. It is generally believed that four antigenic sites of HA’s- the Ca, Cb, Sa and Sb, represent the hot-spot for mutations that affect the receptor binding site, and consequently contribute to antigenic variability, receptor binding and preferences, functionality of virus-host cell fusion, and subsequent virulence^23^. These dynamic issues have raised concerns about an acquired resistance, reduced effectiveness of currently available anti-influenza A drugs^24–26^, and also highlights the significance of gaining better understanding of the nature of mutations, in the context of evolutionary conservation amidst circulating influenza strains.

Studying the evolution, genomics, and structural alterations in Influenza A (H1N1) strains are essential for a better understanding of its diversifications, emergence, virulence and resistance. In the present study we have performed a detailed genetic and structural analysis of the HA and NA proteins of influenza (H1N1) strains isolated during the period 2009–2017. Phylogenetic analysis of HA and NA gene sequences showed a chronological and similar clustering pattern with globally circulating strains. For further genetic analysis of H1N1 strains, we studied the positions of identified mutations on the HA and NA protein structure. We hope that knowledge gained in this regard might help the scientific community comprehend these mutations that have taken place in various influenza strains circulating globally and design inhibitors to effectively eradicate the infection.

## Results and Discussion

During the period of 2009–2017, a total of 5,555 oropharyngeal swabs were collected from patients hospitalized with acute respiratory illness at various hospitals in Kerala (Supplementary Fig. S1). Real-time PCR-based analysis resulted in 1,520-positive cases for influenza A (H1N1) virus. Among the H1N1 positive cases, 743 (48.88%) were males and 777 (51.12%) were females (Supplementary Fig. S2). The age-wise analysis suggests that all age groups were affected with maximum positivity in 18–35 years (43.22%) age group and minimum positivity in <1 year group (0.92%). The median age of patients with influenza A virus infection was 28 (range, below 1 yr – 55 + yr) years (Supplementary Fig. S3). Monthly analysis of data showed that influenza A H1N1 primarily circulated from May to August during the period of 2009–2017 (Supplementary Fig. S4). In the current study, we aim to analyze the molecular evolution and genetic characterization of HA and NA genes of circulating influenza A (H1N1) subtype since August 2009 until April 2017.

### Evolutionary characteristics of H1N1 viruses

The mean evolutionary rate of the HA and NA genes of H1N1 strains analyzed in this study exhibited an evolutionary rate of 5.16 × 10^−3^ and 4.27 × 10^−3^substitutions/site/year, respectively. The evolutionary rates noticed for H1N1 strains analyzed in our study are comparable to previous reports^27^, suggesting a relative consistent evolutionary rate of the H1N1 strains. The Tajima’s D statistic for full-length sequence alignment suggested a strong purifying selection during the evolution of influenza A (H1N1) in India (*D* value of −1.685580 for *HA* and −2.110059 for *NA*).

**Figure 1:**
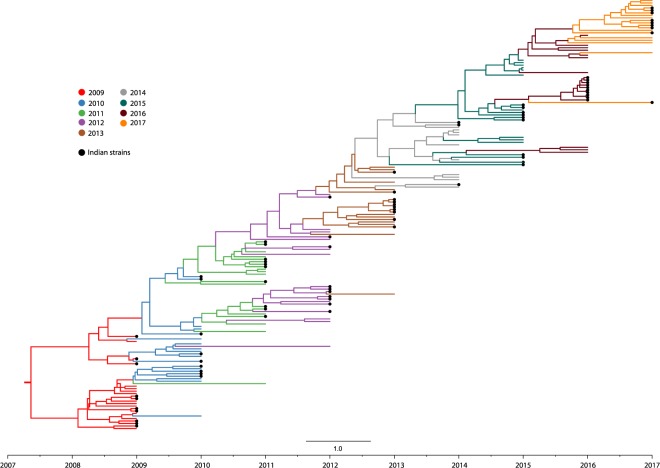
Phylogenetic tree of H1N1 influenza A virus from Indian and global strains reported from 2009 till 2017 with branches colored by year of isolation. Phylogenetic tree of HA gene from 82 Indian and 90 global strain was reconstructed using Bayesian Markov Chain Monte Carlo (MCMC) approach using BEAST v.2.5.2^41,42^ and divergence time estimation using an exponential relaxed clock model. The trace file was visualized using TRACER v.1.7.1^43^.

To better understand the evolutionary trend of Influenza A (H1N1) viruses in India during the period of 2009–2017, HA sequences of strains isolated from our study (Supplementary Table S1) were analyzed along with the locally isolated Indian and global strains (Supplementary Table S2). The phylogenetic tree of HA genes were reconstructed using Bayesian Markov Chain Monte Carlo (MCMC) approach and divergence time estimation using an exponential relaxed clock model^28–30^. The Maximum Clade Credibility tree produced by the Bayesian methodology reveals a temporal pattern of H1N1 evolution, which is congruent with its isolation period (Fig. 1). Additionally, the HA of Indian strains found to have similar mutations circulating worldwide and hence, exhibited a similar clustering of Indian isolates with globally isolated strains in the phylogeny (Fig. 1; Supplementary Fig. S5), a similar pattern reported in the World Health Organization annual reports since 2009 until 2017^31^. H1N1 viruses circulating in India during the period 2009–2010 (Supplementary Table S3) clustered within clades1, 3, 4 and 7 (Supplementary Fig. S6). Following 2011, majority of H1N1 viruses belonged to the pre-established clade 6 and its sub-clades 6A, 6B, 6C and 6B.1. It was observed that all the 2017 H1N1 strains falls into the sub-clade 6B.1 and was genetically similar to the 2017–2018 vaccine strain A/Michigan/45/2015 (Supplementary Fig. S6). Similar to HA, phylogenetic analysis of NA sequences was performed using the Maximum Likelihood method implemented within the MEGAX^32^ software package (Supplementary Table S4; Supplementary Fig. S7). We also observed that the major clades of the phylogeny are structured in a temporal manner and the Indian strains were clustering with H1N1 strains circulated worldwide of the same year.

### Distinct genetic and structural variations in the HA domain

Alignment of HA amino acid sequences of influenza A (H1N1) viruses isolated during 2009–2017 period showed several amino acid substitutions when compared to reference strain (A/California/04/2009) and vaccine strain (A/California/07/2009). In total, 18 substitutions were reported in the HA protein; five of the identified mutations were found in antigenic sites and one in the receptor binding site (Table 1). The H1N1 strains circulating in India since 2009 are characterized by the mutations P100S/S220T/I338V i.e., possible beneficiary mutations that got fixed in the circulating strains. During 2010, no additional variants were encountered in H1N1 viruses, instead previously reported mutations circulated with variable frequencies. In 2011, substitution E391K was introduced in the viral population and became fixed in the subsequent seasons. It was observed that despite the minor influenza H1N1 activity during 2012, new viruses evolved carrying additional substitutions S202T and S468N. Previous studies have reported that S202T substitution would increase the receptor-binding avidity^33^. During 2013, three additional substitutions D114N/K300E/E516K were observed in majority of viruses and became characteristic for the subsequent influenza seasons. Viruses isolated during 2014 season were found to harbor two new mutations K180Q and A273T. Importantly, the mutation K180Q, discriminated strains that circulated before 2013 from those that circulated after 2013 (Table 1). In 2015, India experienced an unpredictable increase in influenza H1N1 activity and observed two substitutions A13T and S101N, apart from the fixed mutations that have been established in previous seasons. It was also noticed that mutation T214A present in >90% viruses during the 2009 pandemic period disappeared in 2010 to 2014 periods but reappeared in 2015 (Table 1). Presence of T214A substitution in HA genes was reported to decrease binding avidity^33^. In 2016, majority of H1N1 viruses were characterized by two variants, R62K and V537A, which gradually got lost in 2017. Viruses circulated during 2017 were detected with four additional substitutions I233T, S179N, S181T and I312V among which S181T and I312V were found to be unique mutations in Indian isolates when compared with globally circulating strains (Table 1).

**Table 1:** Mutations in the HA protein of Influenza A (H1N1) strains during the period of year 2009–2017 as compared to the reference strain A/California/04/2009 and vaccine strain A/California/07/2009.

Year 2009	Year 2010	Year 2011	Year 2012	Year 2013	Year 2014	Year 2015	Year 2016	Year 2017
						A13T	A13T	A13T
							**R62K**	
P100S	P100S	P100S	P100S	P100S	P100S	P100S	P100S	P100S
						S101N	S101N	S101N
				D114N	D114N	D114N	D114N	D114N
								S179N^**a**^
					K180Q^**a**^	K180Q^**a**^	K180Q^**a**^	K180Q^**a**^
								**S181T** ^**a**^
			S202T^**b**^	S202T^**b**^	S202T^**b**^	S202T^**b**^	S202T^**b**^	S202T^**b**^
T214A						T214A	T214A	T214A
S220T^c^	S220T^**c**^	S220T^**c**^	S220T^**c**^	S220T^**c**^	S220T^**c**^	S220T^**c**^	S220T^**c**^	S220T^**c**^
								I233T^**d**^
					A273T	A273T		A273T
				K300E	K300E	K300E	K300E	K300E
								**I312V**
I338V	I338V	I338V	I338V	I338V	I338V	I338V	I338V	I338V
		E391K	E391K	E391K	E391K	E391K	E391K	E391K
			S468N	S468N	S468N	S468N	S468N	S468N
				E516K	E516K	E516K	E516K	E516K
							**V537A**	

Only those mutations which are found universally or >90 percent of the Indian sequences are included here. Amino acid mutations that are unique to Indian strains identified by comparing with global strains are highlighted in bold.

^a^Mutations found in the Sa antigenic site, ^b^Mutations found in the Sb antigenic site, ^c^Mutations found in the Ca antigenic site, ^d^Mutations found in the receptor binding site, Bold – Mutations unique to Indian strains.

**Figure 2:**
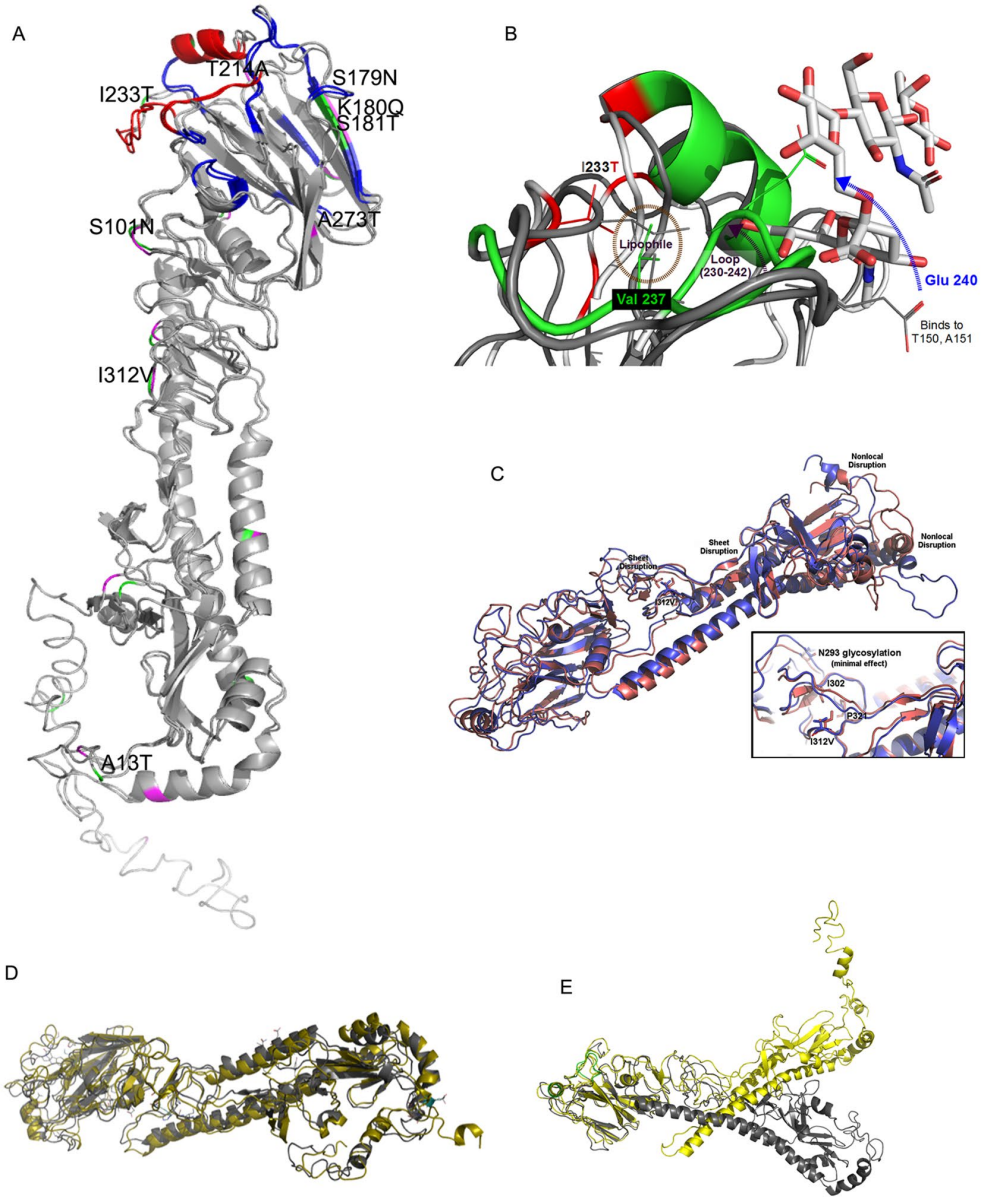
Three-Dimensional Structural and Molecular Dynamic Simulation Analysis of HA protein. (**A**) Superimposed structures of 2009 pandemic H1N1 HA, PDB: 3UBE and mutated HA, where binding site residues and the antigenic site residues are highlighted in red and blue, respectively. The mutated residues are highlighted in green in the mutated and purple in the 2009 pandemic H1N1 HA structure. (**B**) Conformational shift due to single substitution I233T on HA domain. (**C**) Conformational deviation between the wild-type and isoleucine to valine substitution at 312 position in the HA domain was found to disrupt an important Ile312- Ile302 interaction which led to unraveling of the small beta sheet which Ile302 is part of, which in-turn, destabilized a second sheet (further right in the graphic) which ultimately induced substantial nonlocal within the C-terminal domain (far right in the graphic). (**D**,**E**) Root-mean-squared-deviations in structure of 2017 isolate (A/Kerala/RGCBH5/2017) with respect to analogous HA 2009 structure in medium-dielectric media versus high dielectric scenario. Grey is medium-dielectric; yellow is high-dielectric. (**D**) HA domain of 2017 isolates (A/Kerala/RGCBH5/2017). (**E**) HA domain of analogous A/California/2009.

A homology based modeled HA mutated structure in comparison with California 2009 reference strain revealed that the noted mutations in circulating influenza A(H1N1) strains are not likely to alter the overall 3D tertiary structure of HA (RMSD = 0.986) (Fig. 2A; Supplementary Fig. S8). Substitutions at positions P100S, S101N, D114N, S179N, K180Q, S181T, S202T, T214A, S220T and I233T were more frequently found in the head domain of H1N1 viruses (Supplementary Fig. S8) including four changes in the antigenic binding site (S179N, K180Q and S181T-Sa; S202T-Sb; S220T-Ca) and one (I233T) in the receptor binding site. In the mutant K180Q, the side chain of Lys was involved in C-H–Π interactions through its CH2 moiety with aromatic ring of F134 and W140. The amino group of Lys was also involved in N-H–Π/cation–Π interactions with the aromatic rings. However, in the mutated protein side chain of Gln through its amide group interacted with the aromatic ring of the side chain of F134 resulting in Π-Π stacking interaction, leading to no effect on the secondary structure. To determine the impact of the noted mutations on their functionality, we next performed structural analysis of these mutations using molecular dynamic simulation. We hypothesize that the functional effects of these mutations in and around the binding site might destabilize or alter its orientation to the incoming glycan ligand or trigger conformational changes on ligand binding which might be important for its virulence.

### Impact of I233T mutation on glycan specificity

A single substitution I233T in the HA domain could possibly diminishes its pH stability by virtue of disrupting a lipophilic contact between the I233 and V237 (Fig. 2B). Such an intra-molecular change is expected to modify the conformation of a loop formed by amino acids 230–242, a component of the glycan binding site. The outlook of the backbone conformation predicted to trigger a slight broadening of the active site, which would normally reduce receptor specificity. However, such a conformational shift will also interfere with an electrostatic stabilizing between E240 and a pair of H-donors (T150 and the backbone amide of A151). As is shown in Fig. 2B, these coordinated intra-molecular alterations - which we noticed in all of the 2017 Indian H1N1 isolates, will veer out the E240 into space normally used for the glycan binding. In addition, the flexibility of the E240 glutamate side chain could also admit a glycan, and thus, a highly polar side chain of this nature is rather likely to influence binding kinetics.

### S181T and I312V mutations in the HA domain leads to cascading structural-functional implications

The S181T and I312V mutation was found to be unique to H1N1 strains isolated from India. Structural analysis of the antigenic region revealed that S181T mutation, found among all analyzed 2017 Indian isolates, will disrupt a side-chain beta sheet H-bond stabilization and in-turn, might cause the two-stranded sheet to decouple or shift. Although the noted two-stranded sheet is not located close to any glycosylation sites, a sheet disruption is likely to have cascading structural implications through the rest of the protein as observed in our dynamic simulations. The mutation I312V also observed in all the 2017 isolates may destabilize a small hydrophobic knot (I312, I303, and P321) in close vicinity to the N293 glycosylation site, and this may have consequences to glycosylation propensity. An isoleucine to valine mutation leads to a fairly subtle decline in lipophilicity, but for a knot as small as the one observed, is expected to be sufficient to shift the structure. Conformational deviation between the wild-type and I312V HA, as derived from accelerated molecular dynamic simulations (Fig. 2C) indicated that the isoleucine to valine mutation did neither dissipate the small hydrophobic knot nor alter the presentation of the Gln 293 glycosylation site. However, surprisingly, the isoleucine to valine substitution disrupted an important Ile312-Ile302 interaction which led to unraveling of the small beta sheet which Ile302 is part of, which in-turn, destabilized a second sheet (Fig. 2C,further right in the graphic), leading to an induced substantial nonlocal within the C-terminal domain (Fig. 2C, far right in the graphic). We suggest that I312V mutation will have a substantial effect either on the viability of the trimer, or (perhaps more likely) the ultimate conformation of the trimer.

### pH-dependent structural flux in the HA protein

Molecular dynamic simulation of the HA domain in medium-dielectric media versus high dielectric scenarios in the 2017 H1N1 isolates from India revealed that potential pH effects of the HA domain are appreciably higher for the 2017 isolates (between pH 2.950–6.109) relative to the 2009 isolates (pH2.798). Lower root mean square values reflect predictions with caution due to a small number of 2017 H1N1 isolates here, for a better pH stability and an effective denaturation. However, consequently, it is also possible that under a pH-stress, the structures of these isolates might adapt to a novel structure that could conceivably be somehow advantageous for viral survival or infectious prospects. These observations also suggest that adaptation to separate hosts requires an optimal pH of membrane fusion. It is possible that a HA mutant with a higher pH of fusion might replicate efficiently than others. In this context, a closer analysis of the structural changes in the 2017 isolates relative to 2009 isolates in high-RMSD structures surprisingly revealed that a pH-dependent structural flux is advantageous for the 2017 isolates (Fig. 2D,E). Future validation of these hypotheses is expected to unearth further significance of pH stabilization as one of the primary motivations for the mutations observed in the HA domain in the 2017 isolates.

We next performed a comparable proximity analysis on the instances of anion-anion interactions as mediators of relative pH stability across the different isolates. In contrast, our analysis of the His-Cat stabilization mechanism strongly suggested that the 2017 isolates have adapted their HA domain to attain more effective exploitation (or tolerance) of pH-stress, in-spite of a largely random trend for the anion-anion stabilization. Our analysis also suggested that one or both of the residues (Glu275 and Glu422) are likely to participate in anion-anion interactions in the majority of the 2017 isolates but not in the 2009 isolates, presumably due to positioning in a sensitive location in the 2017 isolates. Note that positions 275 and 422 participate in anion-anion interactions due to conformational shifts manifest is most of the 2017 isolates, and are thus, these observations highlight ‘indirect’ consequences of the noted HA mutations on its functionality. By contrast, the 2009 isolate possesses an anion pair (Asp390-Glu391) that disappears in the 2017 isolates as a direct consequence of the E391K mutation manifest in all 2017 forms of HA.

### Distinct genetic and structural variations in the NA domain

The NA protein sequences of H1N1 viruses circulating in India during 2009 till 2017 period were analyzed in comparison to NA of vaccine strain A/California/07/2009. During the early pandemic period, we also noted that several defining mutations (V106I & N248D) in the NA domain became fixed - similar to HA domain. Most of the amino acid substitutions observed during the pandemic period did not persist into the late pandemic phase and several new mutations, such as V241I and N369K, observed to be dominating in the late phase of the pandemic in the NA protein (Supplementary Table S5). Sequence analysis indicated that the 17 mutations in the NA domain have occurred at various time points in the 2009 H1N1 lineage (Supplementary Table S5). Interestingly, the H275Y and N295S mutations in Influenza A (H1N1) strains have been previously shown to be associated with multiple drug resistance^34^; however, these mutations were not observed in H1N1 isolates analyzed in this study. Similarly, we did not notice previously found NA mutations at position I223, H275, Q313 and I427 which are responsible for binding with neuraminidase inhibitors^35–38^.

**Figure 3:**
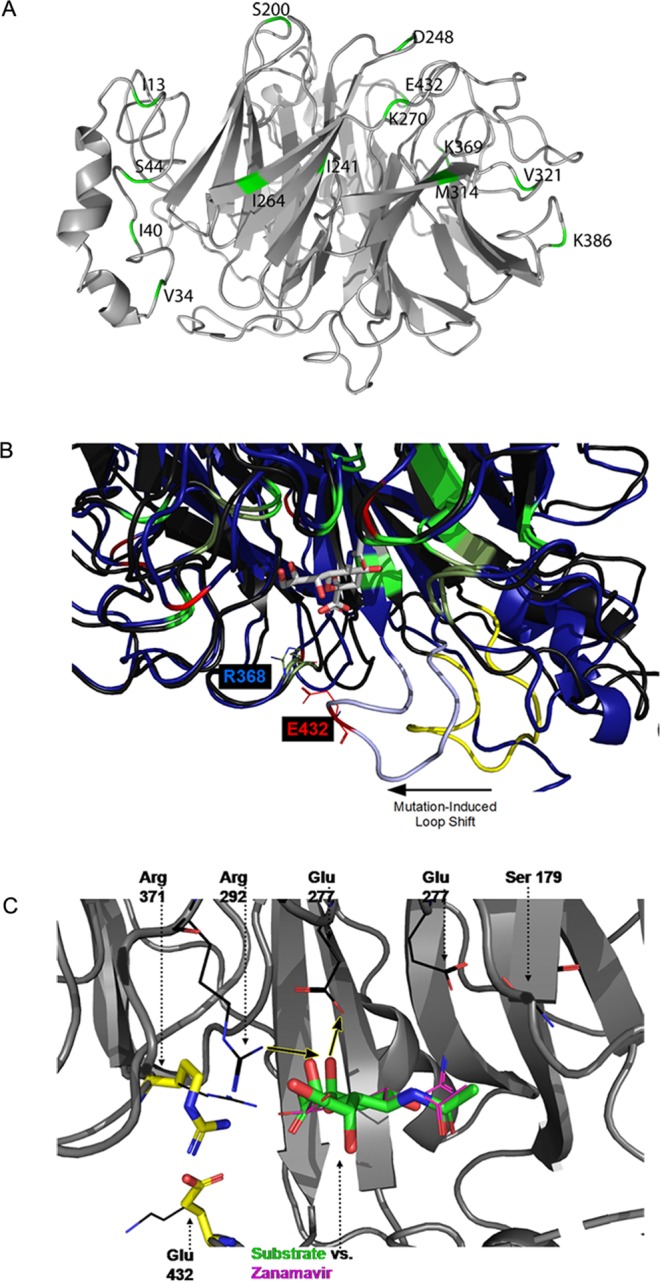
Three-Dimensional Structural and Molecular Dynamic Simulation Analysis of NA protein. (**A**) Superimposed structures of 2009 pandemic H1N1 NA, PDB: 3TI6 and mutated NA. The mutated residues are highlighted in green. (**B**) Effect of mutation K432E on neuraminidase active site reveal that the K432E mutation induces a major loop shift in the vicinity of the active/catalytic site, narrowing the cavity due to the formation of a new salt bridge between E432 (mutated) and R368 residue. (**C**) Effect of the presence of an additional hydroxyl leading to compensatory substrate interactions with E277 and R292. And formation of an E432/R371 salt-bridge is found to deprive the inhibitors of a key electrostatic pharmacophore feature (R371 cationic side chain binding to ligand carboxylate).

Based on size, shape and nature of the side chains, the mutations in the NA domain could be grouped as Group1: I34V, L40I, V241I, V264I and I321V; Group2: N44S, N200S; N270K, N369K, N386K and K432E. Group1 NA mutations involved hydrophobic amino acids contain isoleucine, present either in the loops or the β-strands, and predicted to have no effect on the secondary structural elements. Group 2 NA mutations N44S, N200S were between the polar and neutral molecules but differ in the size of side chain; N to K or K to E substitutions was found to be on the surface of the loops. It is apparent from the molecular view that the residue 20–27 could acquire a helical structure completely in the mutated protein. In the mutated structure most of the residues in this segment were found to have torsion angles corresponding to ϒ-turn. The origin of this complete conversion to helical form arises due to the mutation I34V, due to steric relaxation and onset of hydrophobic interactions between the non-polar side chains. Comparison of the homology modeled mutated NA structure with the reference revealed that the mutations are not effecting the overall 3D structure of the proteins (RMSD = 0.486) (Fig. 3A).

Further structural analysis using molecular dynamic simulation showed that K432E mutation in the NA domain enables a new salt bridge near neuraminidase active site. We found that the K432E mutation induces a major loop shift in the vicinity of the active/catalytic site, narrowing the cavity due to the formation of a new salt bridge between E432 (mutated) and R371 (Fig. 3B) in all of the 2017 isolates except one RGCB2017 isolate. Although inhibitor’s core dihydro-4H-pyran-2-carboxylic acid is smaller than the substrates’ core 2-hydroxyoxane-2-carboxylic acid, the inhibitors are volumetrically roughly the same size as the smallest conceivable substrate, and therefore, pharmacophore aspects relating to the amine/guanidine are likely to play an important role in these conformational changes. In terms on functional impact of the K432E relative to modulating the effect of Zanamavir or Oseltamavir, these observations suggest that the formation of an E432/R371 salt-bridge will deprive the inhibitors of a key electrostatic pharmacophore feature (R371 cationic side chain binding to ligand carboxylate). Nominally, the salt-bridge is expected to also deprive the substrate of this interaction. However, the greater substrate ring saturation (chair, rather than strained chair) and the presence of an additional hydroxyl group will lead to a compensatory substrate interaction with E277 and R292 (Fig. 3C), preventing availability of these residues for the inhibitors. It is anticipated that such a mutation might have a disproportionate effect on the binding kinetics of the neuraminidase inhibitors, and thus, impair the effectiveness of the currently used therapeutic inhibitors.

In conclusion, our study reveals evolutionary relationship of H1N1 strains circulating in India with global strains during the post pandemic period. In addition, the mutational, structural and functional analysis indicate that the regionally acquired mutations in the HA and NA domains may be associated with a needed adaptability for sustenance locally for an efficient human transmissibility. In India the last two years, an unprecedented increase in the number of influenza A virus infection have been detected in the human population that may be a reflection of better detection technologies or a need of constant surveillance to monitor any changes in the influenza A viruses that could increase their potential for transmission and virulence in the human population. The findings presented here offer a better insight for developing the next generation of spatially distinct therapeutic inhibitors for accounting the observed mutations in the circulating isolates. In addition, results presented here provide a rationale to undertake a broader study involving an approach to design the next generation of effective molecules – in addition to providing generic tools to conduct a predictability screening the affected population to the available agents.

## Methods

### Sample collection

From August 2009 till April 2017, five thousand five hundred and fifty five oropharyngeal swab samples collected from patients hospitalized with acute respiratory illness at various hospitals in Kerala were referred to the diagnostic facility of Rajiv Gandhi Centre for Biotechnology (RGCB), Kerala, India, in cold chain for H1N1 diagnosis.

### Molecular detection, gene amplification and sequencing

RNA was extracted from clinical samples using the QIAamp® Viral RNA Mini Kit (Qiagen) according to the manufacturer’s instructions. The *in-vitro* qualitative detection of H1N1 virus from respiratory specimens was performed using Real Star® Influenza Real Time PCR Kit 3.0 (Altona diagnostics GmbH, Germany). Positive samples for those with Ct values ≤ 30 were grown in MDCK cells for one passage. Virus titer was confirmed by hemagglutination assay (HA) and isolates showing HA titer ≥40 were further selected for HA and NA gene amplification and sequencing.

Full length of 43 HA and 23 NA gene segments (Supplementary Table S1) were amplified by reverse transcriptase polymerase chain reaction (RT-PCR) using Superscript III RT-PCR system (Invitrogen Corporation, USA) and the primer pairs recommended by the World Health Organization, USA^39^. The amplified product size was verified on 1% agarose gel and amplicons were PCR purified using Nucleo Spin® Gel and PCR clean-up kit (MACHERRY-NAGEL GmbH & CoKG, Germany). Sequencing was carried out using M13-forward and M13-reverse primers, using the Big Dye terminator V3.1 cycle sequencing kit (ABI, Foster City, CA) and processed for capillary electrophoresis on an ABI 3500 DNA analyzer. Data analysis was done using Sequence Scanner Software version 2 and Bio Edit version 7.2.5. Both HA and NA sequences from this study were deposited into GenBank (Supplementary Table S1). Clinical sample processing, virus culture and RNA isolation were done using recommended biosafety measures in a BSL-2 laboratory.

### Data collection and phylogenetic analysis

For analysis on molecular evolution of influenza A H1N1 both nucleotide of full-length HA and NA genes of influenza A(H1N1)strains isolated in India and globally were downloaded from NCBI Influenza Virus Resource database (https://www.ncbi.nlm.nih.gov/genomes/FLU/Database/nph-select.cgi). From each year, from the large collection of global isolates, 10 sequences were randomly selected along with 43 HA and 23 NA sequences isolated in RGCB, Kerala during 2009–2017. A total 82 HA and 56 NA sequences from Indian isolates and 89 HA and 90 NA sequences from global strains were sampled independently for our phylogenetic analysis. The multiple sequence alignment of Indian and global HA and NA sequences were done using clustalW in MEGA X^32^. Initial Maximum-likelihood (ML) phylogenetic analysis was conducted using Tamura3-parameter model in MEGA X^32^. JModelTest^40^ was used to find out the best substitution model for Bayesian analysis and this analysis resulted in the “TPM1uf + G” model as the best fit for the data. The BIC score of the models tested in the present study are listed in Supplementary Table S6. Bayesian MCMC analysis was performed using BEAST v.2.5.2^41,42^ with “TPM1uf + G” as the substitution model along with a relaxed exponential clock across branches, the chain length is set as 100 Million steps and for every 10000 chains samples are logged. Sample isolation dates were used for calibrating the tree. TRACER v.1.7.1^43^ was used to visualize the results and convergence was assessed with the effective sample size values after removing 10% of the iterations as burn-in. Maximum clade credibility tree was generated using Tree Annotator and visualized using Fig tree^44^.

### HA and NA sequence characterization

A comprehensive dataset of 15,215 Hemagglutinin (HA) and 3,874 Neuraminidase (NA) amino acid sequences of Influenza A (H1N1) viruses isolated globally between 2009 and 2017 were downloaded from NCBI influenza database^45^. Among these, 443 HA and 187 NA sequences were of Indian strains. Given such a large dataset, we re-sampled one sequence per country per year from non-Indian samples. A/California/7/2009 and A/California/4/2009 were used as the reference strains in this study. There was no NA sequence deposited from India for the year 2016 in NCBI database hence could not be included in the analysis. Year wise HA and NA amino acid sequences from Indian strains were aligned with its homolog’s from various countries using MAFFT^46^ program. The alignment was optimized using the global parameters (Gin-si) with 1,000 iterates and visualized using JALview^47^. A mutation was considered as widely distributed only if it were present in more than 90 percent of Indian strain sequences.

### 3D Structural Analysis of HA and NA Proteins

To ascertain whether the mutations alter the structure of the HA and NA proteins, they were modeled using I-TASSER^48^ and energy minimization was done using YASARA^49^ server. Pymol^50^ software was used for 3-dimensional structure visualization and structural similarity estimation (Lushington*inSilico* Consulting, Kansas, USA). For conformational stability and variation predictions, hemagglutinin and neuramidase 3D structures for common 2009 strains of the H1N1 virus were predicted using default search, threading and optimization schemes available in the I-TASSER server^51^. Such simulations require a definitive amino acid sequence for which, in the case of hemagglutinin, we used the sequence with Uniprot accession C4RTE8, whereas for neuramidase we used the sequence with Uniprot accession C3W5S3. I-TASSER structural prediction for neuramidase produced a c-score of −1.97, and a TM-score of 0.48+/− 0.15, while for hemagglutinin these values were c-score = −0.47, TM-score = 0.65+/− 0.13. Since c-score values typically range from −5.0 to 2.0 (higher is better) and ideal TM-scores are greater than 0.50^52^, it can be concluded that both the predicted neuramidase and hemagglutinin structures are of reasonable quality, although the accuracy of hemagglutinin is likely better. In order to assess the structural implications of observed mutations, the predicted 3D structures were mutationally edited in PyMol^50^, and all native (non-mutated) structures and mutated forms were subjected to 100 ns step Gaussian Accelerated Molecular Dynamics^53^ simulations as implemented in NAMD in an implicit solvent model that mimics the plasma environment (dielectric constant of 80.0). As a measure of verification, the relaxed native structures invariably reproduced to within 3.0 Angstroms that 3D generated by the highly validated I-TASSER protocol.

### Ethics statement

Written informed consent was obtained from all the subjects and in case of anyone below the age of 18 from their legal guardians as well for using the collected samples for diagnostic and research purposes at RGCB. Samples used for research purposes were provided in full confidentiality without any link to personal identity of the subjects. This study was approved by the Institute Human Ethics Committee of Rajiv Gandhi Centre for Biotechnology (IHEC/1/2013/01) and was performed in accordance with relevant guidelines and regulations.

## Supplementary information

Supplementary Information
