# Supplementary material for: Evolutionary, genetic, structural characterization and its functional implications for the influenza A (H1N1) infection outbreak in India from 2009 to 2017
Source: Sci Rep. 2019 Oct 11;9:14690. doi: 10.1038/s41598-019-51097-w (PMC6789102; doi:10.1038/s41598-019-51097-w)
Supplement: Supplementary file 1 — Supplementary Information [file 41598_2019_51097_MOESM1_ESM.pdf]

Supplementary information

Evolutionary, genetic, structural characterization and its functional implications for the influenza A (H1N1) infection outbreak in India from 2009 to 2017

Sara Jones[1#], Shijulal Nelson-Sathi[2#], Yejun Wang[3#], Raji Prasad[1], Sabrina Rayen[2], Vibhuti Nandel[2], Yueming Hu[3], Wei Zhang[4], Radhakrishnan Nair[5], Sanjai Dharmaseelan[6], Dhanya Valaveetil Chirundodh[1], Rakesh Kumar[6*], and Radhakrishna Madhavan Pillai[1*]

[1]Pathogen Biology Program, Rajiv Gandhi Center for Biotechnology, Thiruvananthapuram, Kerala, 695014, India.

[2]Interdisciplinary Biology Program, Rajiv Gandhi Center for Biotechnology, Thiruvananthapuram, Kerala, 695014, India.

[3]Department of Cell Biology and Genetics, School of Basic Medical Sciences, Shenzhen University Health Science Center, Shenzhen, 518060, China.

[4]Shenzhen Gen Read Technology, Shenzhen, 518000, China.

[5]Laboratory Medicine and Molecular Diagnostics Program, Rajiv Gandhi Centre for Biotechnology, Thiruvananthapuram, Kerala, 695014, India.

[6]Cancer Research Program, Rajiv Gandhi Centre for Biotechnology, Thiruvananthapuram, Kerala, 695014, India.

[#]These authors contributed equally to this work.

***Corresponding authors:**
Prof. Radhakrishna Madhavan Pillai, FRCPath, PhD, FASc, FNASc, FAMS, FNA.

Email: mrpillai@rgcb.res.in, **Phone**: +91-471-2347-973.

Rakesh Kumar, PhD.
Email: rakeshkumar@rgcb.res.in, **Phone**: +91-471-2781-270.

Supplementary figure S1

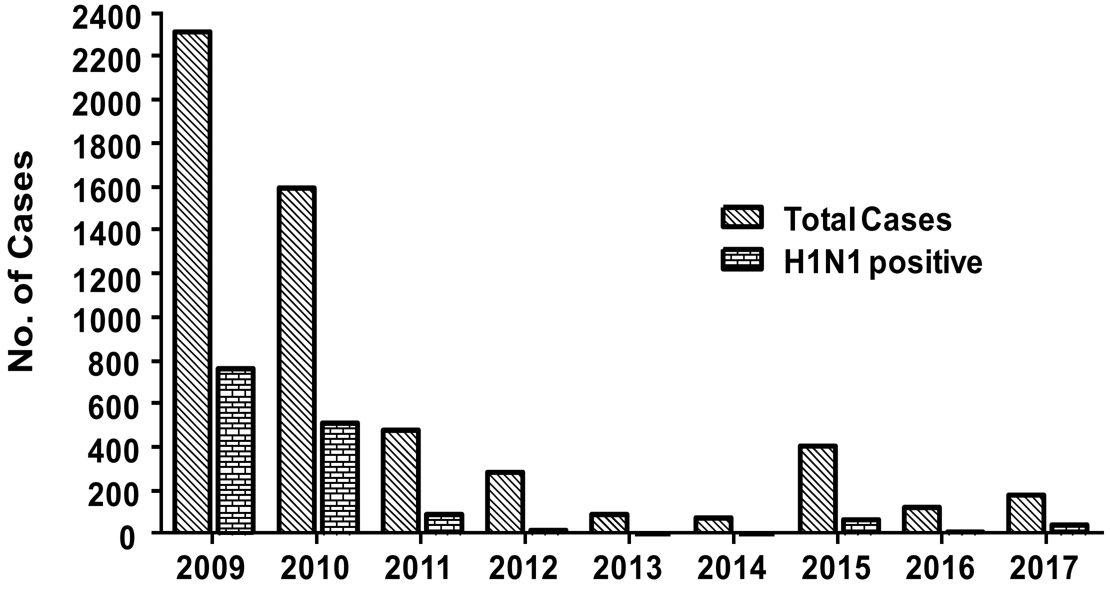

Supplementary Fig. S1. Overall year wise trend ofinfluenza A (H1N1) cases isolated from RGCB, Kerala, India during 2009 – 2017 period.

Supplementary figure S2

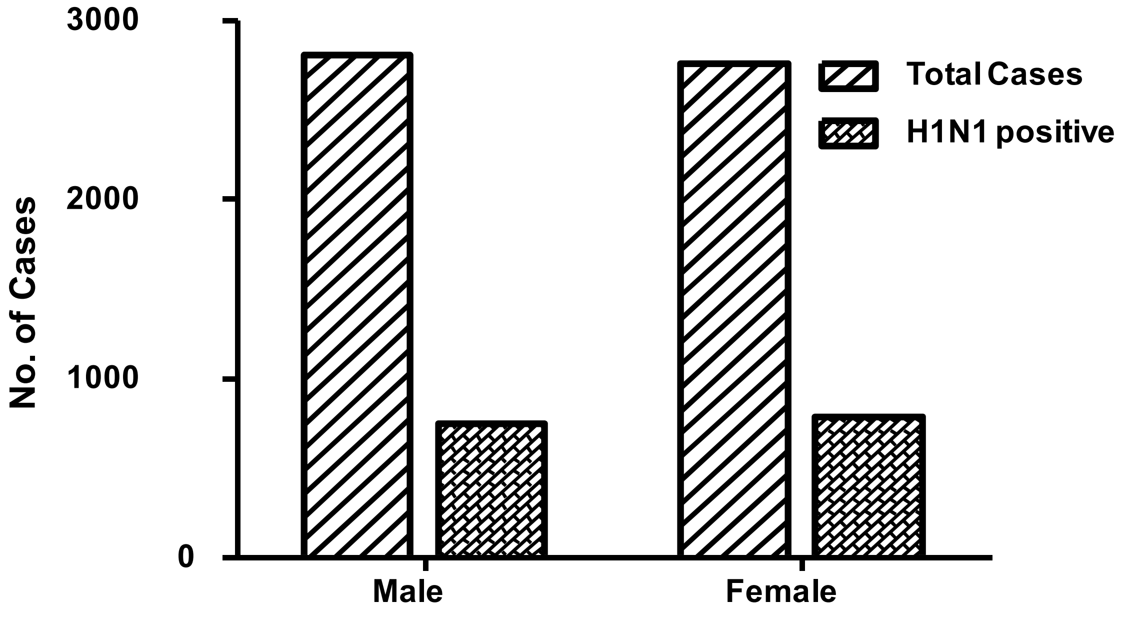

Supplementary Fig. S2. Gender wise distribution of patients infected with influenza A (H1N1) viruses isolated from RGCB, Kerala during 2009 – 2017 period.

Supplementary figure S3

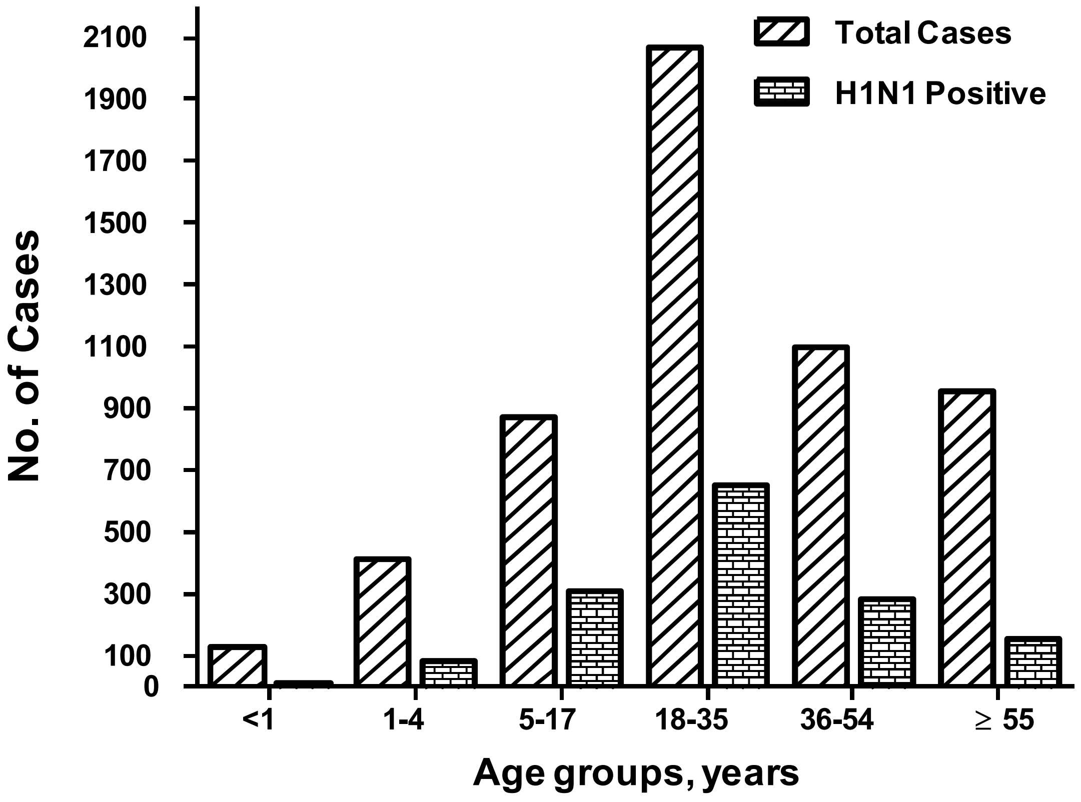

Supplementary Fig. S3. Age wise distribution of patients infected with influenza A (H1N1) viruses isolated from RGCB, Kerala during 2009 – 2017 period.

Supplementary figure S4

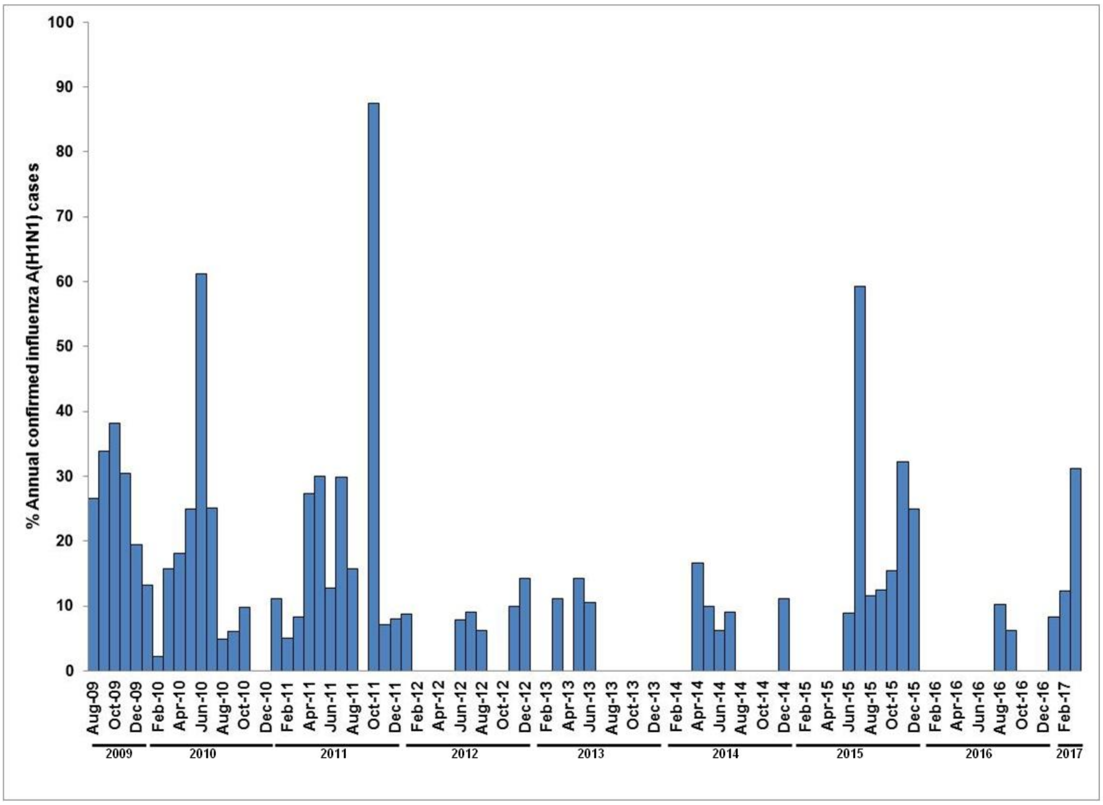

Supplementary Fig. S4. Trends of monthly annual number of positive influenza H1N1 cases isolated from RGCB, Kerala during 2009 to 2017 periods.

Supplementary table S1

Sl.No.	Name of Isolate	Date of Collection	Age	Sex	Gene Sequenced	GenbankID
1	A/Kerala/RGCB_A808/2009	10/02/2009	26yrs	M	HA;NA	MK080167;MK080186
2	A/Kerala/RGCB_A884/2009	10/05/2009	44yrs	M	HA	MK080168
3	A/Kerala/RGCB_A923/2009	10/07/2009	20yrs	M	HA	KM203128
4	A/Kerala/RGCB_B172/2009	10/14/2009	9yrs	M	HA;NA	MK080169;MK080187
5	A/Kerala/RGCB_B205/2009	10/15/2009	26yrs	M	HA;NA	MK080170;MK080188
6	A/Kerala/RGCB_B233/2009	10/16/2009	2 ½ years	M	HA;NA	MK080171;MK080189
7	A/Kerala/RGCB_B234/2009	10/16/2009	6months	F	HA	MK080172
8	A/Kerala/RGCB_B360/2009	10/20/2009	13yrs	M	HA	KM203129
9	A/Kerala/RGCB_C291/2009	12/01/2009	21yrs	F	HA	KM203130
10	A/Kerala/RGCB_C699/2010	03/09/2010	16yrs	F	HA	KM203131
11	A/Kerala/RGCB_C700/2010	03/09/2010	50yrs	M	HA	KM203132
12	A/Kerala/RGCB_C990/2010	06/16/2010	7yrs	M	HA	MK080173
14	A/Kerala/RGCB_C996/2010	06/16/2010	1yr	F	HA	MK080174
15	A/Kerala/RGCB_C998/2010	06/16/2010	18yrs	F	HA	MK080175
16	A/Kerala/RGCB_E658/2011	03/07/2011	44yrs	M	HA	KM203133
17	A/Kerala/RGCB_E679/2011	06/07/2011	18yrs	F	HA	KM203134
18	A/Kerala/RGCB_E680/2011	06/07/2011	21yrs	F	HA	KM203135
19	A/Kerala/RGCB_F2/2011	02/08/2011	4yrs	M	HA	KM203136
20	A/Kerala/RGCB_F294/2012	16/07/2012	24yrs	F	HA	KM203137
21	A/Kerala/RGCB_F302/2012	17/07/2012	69yrs	M	HA	KM203138
22	A/Kerala/RGCB_F311/2012	20/07/2012	1yr	M	HA	KM203139
23	A/Kerala/RGCB_815/2014	01/04/2014	52yrs	M	HA;NA	KY888150;KY888153
24	A/Kerala/RGCB_437/2014	09/06/2014	42yrs	M	HA;NA	KY888151;KY888154
25	A/Kerala/RGCB_344/2015	12/01/2015	53yrs	F	NA	MK080191
26	A/Kerala/RGCB_345/2015	09/01/2015	53yrs	F	HA	MK080176
27	A/Kerala/RGCB_385/2015	14/01/2015	42yrs	M	HA	KY888149
28	A/Kerala/RGCB_546/2015	27/01/2015	70yrs	M	HA	MK080177
29	A/Kerala/RGCB_589/2015	27/01/2015	77yrs	F	HA	MK080178
30	A/Kerala/RGCB_751/2015	03/02/2015	40yrs	F	HA;NA	KY888148;KY888155
31	A/Kerala/RGCB_757/2015	18/06/2015	68yrs	M	NA	MK080192
32	A/Kerala/RGCB_808/2015	03/02/2015	-	M	HA;NA	KU922545;KY888156
33	A/Delhi/RGCB_D10/2015	18/02/2015	-	F	HA;NA	MK080179;MK080193
34	A/Delhi/RGCB_D11/2015	18/02/2015	-	M	HA;NA	MK080180;MK080194
35	A/Delhi/RGCB_D16/2015	25/02/2015	-	M	HA;NA	MK080181;MK080195
36	A/Del/RGCB_D17/2015	28/02/2015	-	F	HA;NA	MK080182;MK080196
37	A/Del/RGCB_D26/2015	28/02/2015	-	F	HA;NA	MK080183;MK080197
38	A/Del/RGCB_D27/2015	28/02/2015	-	F	HA;NA	MK080184;MK080198
39	A/Del/RGCB_D28/2015	28/02/2015	-	M	HA;NA	MK080185;MK080199
40	A/Kerala/RGCB_2047/2017	28/02/2017	73yrs	M	NA	MF510843
41	A/Kerala/RGCB_2108/2017	03/03/2017	18yrs	M	HA;NA	MF510838;MF510842
42	A/Kerala/RGCB_2158/2017	08/03/2017	55yrs	M	HA	MF510835
43	A/Kerala/RGCB_2171/2017	09/03/2017	65yrs	F	HA;NA	MF510836;MF510846
44	A/Kerala/RGCB_2185/2017	08/03/2017	45ys	F	NA	MF510847
45	A/Kerala/RGCB_2247/2017	13/03/2017	33yrs	F	HA;NA	MF510839;MF510844
46	A/Kerala/RGCB_2263/2017	14/03/2017	27yrs	F	HA	MF510840
47	A/Kerala/RGCB_2302/2017	16/03/2017	52	F	HA;NA	MF510841;MF510845
48	A/Kerala/RGCB_2326/2017	21/03/2017	-	M	HA	MF510837

Supplementary Table S1. Details of patients and influenza A (H1N1) viruses isolated during October 2009 to March 2017from RGCB, Kerala and gene segments with genbank accession numbers used in this study*.

*https://www.ncbi.nlm.nih.gov/genbank/

Supplementary table S2

Sl.No.	Name of Isolate	Year of Isolation	Segment	Genbank ID
1.	A/Punjab/007/2009	26/11/2009	HA	KP317211
2.	A/Mumbai/3166/2009	12/09/2009	HA	KM219036
3.	A/Ngp/NIV11203/2009	05/09/2009	HA	CY075892
4.	A/Mumbai/4467/2009	08/12/2009	HA	KM219062
5.	A/Mumbai/3413/2009	19/09/2009	HA	KM219066
6.	A/JammuandKashmir/013/2009	04/12/2009	HA	KP317217
7.	A/Karnataka/001/2009	06/08/2009	HA	KP317205
8.	A/Mumbai/3011/2009	03/09/2009	HA	KM219027
9.	A/Pune/NIV21123/2009	01/12/2009	HA	HM204579
10.	A/Mum/NIV1134/2009	05/08/2009	HA	CY075894
11.	A/JammuandKashmir/021/2010	19/07/2010	HA	KP317225
12.	A/Mumbai/5116/2010	30/07/2010	HA	KM219063
13.	A/Mumbai/4916/2010	08/07/2010	HA	KM219059
14.	A/Bhopal/1544/2010	04/11/2010	HA	KT241016
15.	A/India/NIV38258/2010	02/09/2010	HA	CY084267
16.	A/Chattisgarh/025/2010	07/02/2010	HA	KP317229
17.	A/India/NIV29386/2010	02/06/2010	HA	CY084239
18.	A/Lur/NIV24770/2010	12/02/2010	HA	CY075915
19.	A/India/NIV1028816/2010	17/06/2010	HA	CY084284
20.	A/Kerala/023/2010	19/06/2010	HA	KP317227
21.	A/Bangalore/2327-56/2011	17/11/2011	HA	KR861683
22.	A/Bangalore/1808-53/2011	09/09/2011	HA	KR861681
23.	A/Punjab/041/2011	09/03/2011	HA	KP317245
24.	A/JammuandKashmir/045/2011	31/01/2011	HA	KP317249
25.	A/Bangalore/1326-52/2011	04/08/2011	HA	KR861680
26.	A/Delhi/053/2011	23/06/2011	HA	KP317257
27.	A/Punjab/042/2011	17/03/2011	HA	KP317246
28.	A/Goa/046/2011	23/06/2011	HA	KP317250
29.	A/Punjab/043/2011	28/03/2011	HA	KP317247
30.	A/India/P1114854/2011	23/09/2011	HA	KF280663
31.	A/India/VD122268/2012	07/03/2012	HA	KF280727
32.	A/India/Gwl-06/2012	31/08/2012	HA	KC894815
33.	A/Harda/1718/2012	28/04/2012	HA	KM885032
34.	A/Delhi/068/2012	20/11/2012	HA	KP317272
35.	A/India/P123167/2012	22/03/2012	HA	KX792285
36.	A/Jodhpur/3168/2012	28/09/2012	HA	MF535114
37.	A/Delhi/069/2012	19/12/2012	HA	KP317273
38.	A/India/P122090/2012	07/03/2012	HA	KX792289
39.	A/Bangalore/697-32/2012	12/04/2012	HA	KR861685
40.	A/India/P122707/2012	17/03/2012	HA	KX792266
41.	A/India/P131027/2013	27/01/2013	HA	KF270582
42.	A/Itarsi/2934/2013	15/05/2013	HA	KM885036
43.	A/JammuandKashmir/085/2013	20/02/2013	HA	KP317289
44.	A/India/P131845/2013	15/02/2013	HA	KF280743
45.	A/Haryana/081/2013	22/01/2013	HA	KP317285
46.	A/Uttrakhand/083/2013	22/02/2013	HA	KP317287

47.	A/Delhi/087/2013	17/02/2013	HA	KP317291
48.	A/India/Pun1312095/2013	29/07/2013	HA	KR052553
49.	A/India/Nag1320058/2013	19/07/2013	HA	KR052535
50.	A/India/Pun1318508/2013	10/09/2013	HA	KR052565
51.	A/India/Pun14549/2014	18/01/2014	HA	KR052610
52.	A/India/Che147504/2014	14/03/2014	HA	KR052618
53.	A/India/Pun14584/2014	21/01/2014	HA	KR052611
54.	A/India/Kol-4632/2015	01/02/2015	HA	KU695607
55.	A/India/Pun153389/2015	27/02/2015	HA	KR052632
56.	A/India/Pun151268/2015	27/01/2015	HA	KR052627
57.	A/India/Pun151508/2015	03/02/2015	HA	KR052631
58.	A/India/Pun153388/2015	27/02/2015	HA	KR052633
59.	A/India/Kol-4651/2015	01/02/2015	HA	KU695608
60.	A/India/DRDE_GWL703/2015	07/03/2015	HA	KT867221
61.	A/India/Kol-4628/2015	01/02/2015	HA	KU695606
62.	A/India/DRDE_GWL84/2015	17/03/2015	HA	KX078485
63.	A/India/DRDE_GWL721/2015	08/03/2015	HA	KT867223
64.	A/Assam/RMRC_711/2016	13/07/2016	HA	MF564211
65.	A/Assam/RMRC_693/2016	06/07/2016	HA	MF564210
66.	A/Assam/RMRC_605/2016	27/06/2016	HA	MF564206
67.	A/Assam/RMRC_449/2016	06/06/2016	HA	MF564213
68.	A/Assam/RMRC_226/2016	16/03/2016	HA	MF564209
69.	A/Assam/RMRC_609/2016	27/06/2016	HA	MF564207
70.	A/Assam/RMRC_494/2016	13/06/2016	HA	MF564203
71.	A/Assam/RMRC_709/2016	13/07/2016	HA	MF564208
72.	A/Assam/RMRC_598/2016	24/06/2016	HA	MF564205
73.	A/Assam/RMRC_527/2016	21/06/2016	HA	MF564204
74.	A/Mysore/MCVRAF7736/2017	25/03/2017	HA	MG572209
75.	A/Shimoga/MCVRAF9709/2017	2017/04/24	HA	MG572215
76.	A/Kolar/MCVRAF3154/2017	27/01/2017	HA	MG572212
77.	A/Mysore/MCVRAF7729/2017	25/03/2017	HA	MG572208
78.	A/Chikmagalur/MCVRAF7881/2017	30/03/2017	HA	MG572211
79.	A/Ernakulam/MCVRAF7821/2017	29/03/2017	HA	MG572210
80.	A/Calicut/MCVRAF9834/2017	24/04/2017	HA	MG572216
81.	A/Nilgiris/MCVRAF7809/2017	28/03/2017	HA	MG572214
82.	A/Mysore/MCVRAF7638/2017	25/03/2017	HA	MG572213
83.	A/England/403/2009	01/06/2009	HA	CY070721
84.	A/NewYork/7036/2009	16/12/2009	HA	CY062002
85.	A/Scotland/Glasgow_413608/2009	03/06/2009	HA	CY107607
86.	A/Mexico/24060/2009	28/04/2009	HA	CY147939
87.	A/RiodeJaneiro/5007/2009	23/07/2009	HA	KC967087
88.	A/Osaka/2/2009	16/05/2009	HA	GQ219579
89.	A/WestVirginia/13/2009	25/08/2009	HA	KC780679
90.	A/Arizona/17/2009	15/10/2009	HA	KC782056
91.	A/HongKong/H090-793-V20/2009	14/08/2009	HA	CY120485
92.	A/Mexico/24050/2009	27/04/2009	HA	CY147867
93.	A/Singapore/GP4363/2010	25/10/2010	HA	JX309893
94.	A/Sydney/DD3-01/2010	19/09/2010	HA	CY092430
95.	A/England/04860493/2010	28/11/2010	HA	JX625726
96.	A/SaoPaulo/29269/2010	03/05/2010	HA	HQ595292

97.	A/Taiwan/65642/2010	01/05/2010	HA	JN381270
98.	A/Chile/115/2010	01/01/2010	HA	CY093205
99.	A/Hawaii/06/2010	19/04/2010	HA	KC782144
100.	A/Delaware/05/2010	10/11/2010	HA	KC882120
101.	A/Qingdao/220/2010	2010/03/01	HA	CY067630
102.	A/England/283/2010	06/12/2010	HA	LN867708
103.	A/SantaCruz/7941/2011	29/09/2011	HA	KF612082
104.	A/shandong-fushan/SWL172/2011	01/04/2011	HA	MG982581
105.	A/Thailand/KS10403/2011	26/04/2011	HA	KP637350
106.	A/Georgia/NHRC0001/2011	27/01/2011	HA	CY092904
107.	A/Tottori/YK041/2011	2011/02/01	HA	AB745403
108.	A/Taiwan/1102/2011	2011/01/19	HA	JN187148
109.	A/Tottori/TT007/2011	01/01/2011	HA	AB745334
110.	A/Tottori/ST652/2011	01/02/2011	HA	AB745320
111.	A/Guangdong/033/2011	28/02/2011	HA	JF929773
112.	A/Vermont/04/2011	14/01/2011	HA	KC881783
113.	A/Ontario/006/2012	02/02/2012	HA	KF551083
114.	A/Texas/24/2012	25/02/2012	HA	KC891569
115.	A/Colorado/14/2012	28/03/2012	HA	KC891306
116.	A/SouthCarolina/3043/2012	04/10/2012	HA	CY130176
117.	A/Washington/24/2012	17/06/2012	HA	KC891401
118.	A/Texas/90/2012	18/10/2012	HA	KF647933
119.	A/Ontario/023/2012	2012/03/13	HA	KF551093
120.	A/Pennsylvania/31/2012	18/12/2012	HA	KF648095
121.	A/NorthCarolina/16/2012	28/03/2012	HA	KC891152
122.	A/Thailand/SR10382/2012	21/08/2012	HA	KP637750
123.	A/Wisconsin/U140093/2013	01/12/2013	HA	KR611200
124.	A/Alaska/07/2013	23/06/2013	HA	KM408937
125.	A/NanPing/SWL1402/2013	06/06/2013	HA	KP019892
126.	A/NanPing/SWL35/2013	18/04/2013	HA	KP019883
127.	A/XiaMen/SWL1123/2013	15/04/2013	HA	KP019882
128.	A/Wisconsin/V140032/2013	01/12/2013	HA	KR611232
129.	A/Massachusetts/01/2013	02/01/2013	HA	KF648105
130.	A/Helsinki/1499/2013	26/03/2013	HA	KM366511
131.	A/Taiwan/80272/2013	13/03/2013	HA	KF495638
132.	A/BritishColumbia/040/2013	20/02/2013	HA	KF761456
133.	A/NewJersey/3622/2014	10/01/2014	HA	CY187290
134.	A/Missouri/03/2014	12/02/2014	HA	KT274520
135.	A/Alabama/3716/2014	06/02/2014	HA	CY187634
136.	A/Tennessee/01/2014	07/01/2014	HA	KM409089
137.	A/NewYork/WC-LVD-14-006/2014	14/01/2014	HA	CY189089
138.	A/Colorado/3558/2014	02/01/2014	HA	CY187226
139.	A/Texas/3676/2014	14/01/2014	HA	CY187344
140.	A/NorthCarolina/3801/2014	06/03/2014	HA	CY187719
141.	A/Helsinki/946M/2014	21/02/2014	HA	KM366767
142.	A/Iowa/27/2014	09/12/2014	HA	KT836443
143.	A/NewMexico/29/2015	12/12/2015	HA	KX004437
144.	A/Michigan/64/2015	04/10/2015	HA	KU509783
145.	A/Adana/05/2015	30/12/2015	HA	KX214548
146.	A/Markazi/0335/2015	22/11/2015	HA	MG230359

147.	A/Colorado/20/2015	22/11/2015	HA	KU509599
148.	A/Jeddah/KFAFH0006/2015	06/11/2015	HA	MF768667
149.	A/Nicaragua/6447_07/2015	17/12/2015	HA	CY256898
150.	A/Wisconsin/90/2015	08/12/2015	HA	KX004388
151.	A/Colorado/35/2015	27/12/2015	HA	KX005050
152.	A/Michigan/63/2015	05/10/2015	HA	KU509657
153.	A/Linkou/0098/2016	02/02/2016	HA	KY615441
154.	A/Oklahoma/12/2016	18/10/2016	HA	CY209528
155.	A/Florida/11/2016	25/01/2016	HA	KX406323
156.	A/Massachusetts/24/2016	11/03/2016	HA	KY044854
157.	A/NewHampshire/04/2016	14/02/2016	HA	KX407283
158.	A/CzechRepublic/14/2016	31/01/2016	HA	KX919244
159.	A/Kentucky/17/2016	11/03/2016	HA	KX410515
160.	A/Arizona/29/2016	19/02/2016	HA	KX408795
161.	A/Montana/23/2016	24/02/2016	HA	KX407683
162.	A/Idaho/01/2017	07/01/2017	HA	CY216945
163.	A/Florida/76/2017	23/10/2017	HA	CY261734
164.	A/SouthCarolina/01/2017	02/01/2017	HA	CY217065
165.	A/California/61/2017	18/07/2017	HA	CY242509
166.	A/Arizona/64/2017	06/11/2017	HA	MG978467
167.	A/Louisiana/68/2017	13/12/2017	HA	MH083648
168.	A/Tunisia/12233/2017	12/12/2017	HA	MG745932
169.	A/Illinois/6472/2017	19/12/2017	HA	MH294838
170.	A/Florida/76/2017	23/10/2017	HA	CY261726
171.	A/Illinois/43/2017	05/12/2017	HA	MH083496

Supplementary Table S2. Influenza A (H1N1) viruses and gene segment from NCBI influenza virus resource database used in this study*.

*https://www.ncbi.nlm.nih.gov/genomes/FLU/Database/nph-select.cgi.

.

Supplementary figure S5

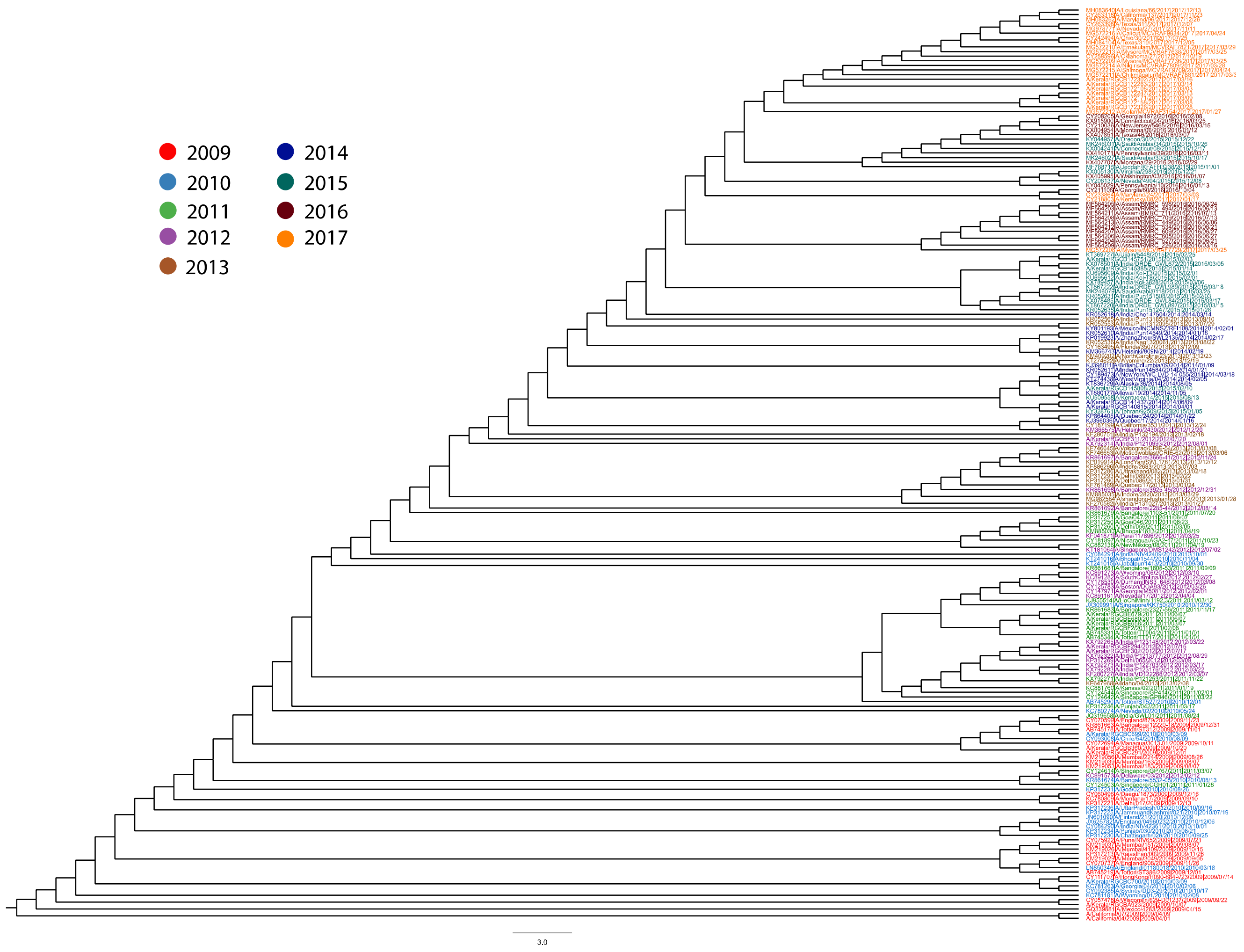

Supplementary Fig. S5. Maximum likelihood phylogeny of HA sequence of H1N1 strains isolated in India and globally during 2009-2017.

Supplementary table S3

Sl.No.	Name of Isolate	Year of Isolation	Genebank ID
1.	A/Punjab/007/2009	26/11/2009	KP317211
2.	A/Mumbai/3166/2009	12/09/2009	KM219036
3.	A/Ngp/NIV11203/2009	05/09/2009	CY075892
4.	A/Mumbai/4467/2009	08/12/2009	KM219062
5.	A/Mumbai/3413/2009	19/09/2009	KM219066
6.	A/JammuandKashmir/013/2009	04/12/2009	KP317217
7.	A/Karnataka/001/2009	06/08/2009	KP317205
8.	A/Mumbai/3011/2009	03/09/2009	KM219027
9.	A/Pune/NIV21123/2009	01/12/2009	HM204579
10.	A/Mum/NIV1134/2009	05/08/2009	CY075894
11.	A/JammuandKashmir/021/2010	19/07/2010	KP317225
12.	A/Mumbai/5116/2010	30/07/2010	KM219063
13.	A/Mumbai/4916/2010	08/07/2010	KM219059
14.	A/Bhopal/1544/2010	04/11/2010	KT241016
15.	A/India/NIV38258/2010	02/09/2010	CY084267
16.	A/Chattisgarh/025/2010	07/02/2010	KP317229
17.	A/India/NIV29386/2010	02/06/2010	CY084239
18.	A/Lur/NIV24770/2010	12/02/2010	CY075915
19.	A/India/NIV1028816/2010	17/06/2010	CY084284
20.	A/Kerala/023/2010	19/06/2010	KP317227
21.	A/Bangalore/2327-56/2011	17/11/2011	KR861683
22.	A/Bangalore/1808-53/2011	09/09/2011	KR861681
23.	A/Punjab/041/2011	09/03/2011	KP317245
24.	A/JammuandKashmir/045/2011	31/01/2011	KP317249
25.	A/Bangalore/1326-52/2011	04/08/2011	KR861680
26.	A/Delhi/053/2011	23/06/2011	KP317257
27.	A/Punjab/042/2011	17/03/2011	KP317246
28.	A/Goa/046/2011	23/06/2011	KP317250
29.	A/Punjab/043/2011	28/03/2011	KP317247
30.	A/India/P1114854/2011	23/09/2011	KF280663
31.	A/India/VD122268/2012	07/03/2012	KF280727
32.	A/India/Gwl-06/2012	31/08/2012	KC894815
33.	A/Harda/1718/2012	28/04/2012	KM885032
34.	A/Delhi/068/2012	20/11/2012	KP317272
35.	A/India/P123167/2012	22/03/2012	KX792285
36.	A/Jodhpur/3168/2012	28/09/2012	MF535114
37.	A/Delhi/069/2012	19/12/2012	KP317273
38.	A/India/P122090/2012	07/03/2012	KX792289
39.	A/Bangalore/697-32/2012	12/04/2012	KR861685
40.	A/India/P122707/2012	17/03/2012	KX792266
41.	A/India/P131027/2013	27/01/2013	KF270582
42.	A/Itarsi/2934/2013	15/05/2013	KM885036
43.	A/JammuandKashmir/085/2013	20/02/2013	KP317289
44.	A/India/P131845/2013	15/02/2013	KF280743
45.	A/Haryana/081/2013	22/01/2013	KP317285
46.	A/Uttrakhand/083/2013	22/02/2013	KP317287

47.	A/Delhi/087/2013	17/02/2013	KP317291
48.	A/India/Pun1312095/2013	29/07/2013	KR052553
49.	A/India/Nag1320058/2013	19/07/2013	KR052535
50.	A/India/Pun1318508/2013	10/09/2013	KR052565
51.	A/India/Pun14549/2014	18/01/2014	KR052610
52.	A/India/Che147504/2014	14/03/2014	KR052618
53.	A/India/Pun14584/2014	21/01/2014	KR052611
54.	A/India/Kol-4632/2015	01/02/2015	KU695607
55.	A/India/Pun153389/2015	27/02/2015	KR052632
56.	A/India/Pun151268/2015	27/01/2015	KR052627
57.	A/India/Pun151508/2015	03/02/2015	KR052631
58.	A/India/Pun153388/2015	27/02/2015	KR052633
59.	A/India/Kol-4651/2015	01/02/2015	KU695608
60.	A/India/DRDE_GWL703/2015	07/03/2015	KT867221
61.	A/India/Kol-4628/2015	01/02/2015	KU695606
62.	A/India/DRDE_GWL84/2015	17/03/2015	KX078485
63.	A/India/DRDE_GWL721/2015	08/03/2015	KT867223
64.	A/Assam/RMRC_711/2016	13/07/2016	MF564211
65.	A/Assam/RMRC_693/2016	06/07/2016	MF564210
66.	A/Assam/RMRC_605/2016	27/06/2016	MF564206
67.	A/Assam/RMRC_449/2016	06/06/2016	MF564213
68.	A/Assam/RMRC_226/2016	16/03/2016	MF564209
69.	A/Assam/RMRC_609/2016	27/06/2016	MF564207
70.	A/Assam/RMRC_494/2016	13/06/2016	MF564203
71.	A/Assam/RMRC_709/2016	13/07/2016	MF564208
72.	A/Assam/RMRC_598/2016	24/06/2016	MF564205
73.	A/Assam/RMRC_527/2016	21/06/2016	MF564204
74.	A/Mysore/MCVRAF7736/2017	25/03/2017	MG572209
75.	A/Shimoga/MCVRAF9709/2017	2017/04/24	MG572215
76.	A/Kolar/MCVRAF3154/2017	27/01/2017	MG572212
77.	A/Mysore/MCVRAF7729/2017	25/03/2017	MG572208
78.	A/Chikmagalur/MCVRAF7881/2017	30/03/2017	MG572211
79.	A/Ernakulam/MCVRAF7821/2017	29/03/2017	MG572210
80.	A/Calicut/MCVRAF9834/2017	24/04/2017	MG572216
81.	A/Nilgiris/MCVRAF7809/2017	28/03/2017	MG572214
82.	A/Mysore/MCVRAF7638/2017	25/03/2017	MG572213
83.	A/Kerala/RGCB_A808/2009	10/02/2009	MK080167
84.	A/Kerala/RGCB_A884/2009	10/05/2009	MK080168
85.	A/Kerala/RGCB_A923/2009	10/07/2009	KM203128
86.	A/Kerala/RGCB_B172/2009	10/14/2009	MK080169
87.	A/Kerala/RGCB_B205/2009	10/15/2009	MK080170
88.	A/Kerala/RGCB_B233/2009	10/16/2009	MK080171
89.	A/Kerala/RGCB_B234/2009	10/16/2009	MK080172
90.	A/Kerala/RGCB_B360/2009	10/20/2009	KM203129
91.	A/Kerala/RGCB_C291/2009	12/01/2009	KM203130
92.	A/Kerala/RGCB_C699/2010	03/09/2010	KM203131
93.	A/Kerala/RGCB_C700/2010	03/09/2010	KM203132
94.	A/Kerala/RGCB_C990/2010	06/16/2010	MK080173
95.	A/Kerala/RGCB_C996/2010	06/16/2010	MK080174

96.	A/Kerala/RGCB_C998/2010	06/16/2010	MK080175
97.	A/Kerala/RGCB_E658/2011	03/07/2011	KM203133
98.	A/Kerala/RGCB_E679/2011	06/07/2011	KM203134
99.	A/Kerala/RGCB_E680/2011	06/07/2011	KM203135
100.	A/Kerala/RGCB_F2/2011	02/08/2011	KM203136
101.	A/Kerala/RGCB_F294/2012	16/07/2012	KM203137
102.	A/Kerala/RGCB_F302/2012	17/07/2012	KM203138
103.	A/Kerala/RGCB_F311/2012	20/07/2012	KM203139
104.	A/Kerala/RGCB_815/2014	01/04/2014	KY888150
105.	A/Kerala/RGCB_437/2014	09/06/2014	KY888151
106.	A/Kerala/RGCB_345/2015	09/01/2015	MK080176
107.	A/Kerala/RGCB_385/2015	14/01/2015	KY888149
108.	A/Kerala/RGCB_546/2015	27/01/2015	MK080177
109.	A/Kerala/RGCB_589/2015	27/01/2015	MK080178
110.	A/Kerala/RGCB_751/2015	03/02/2015	KY888148
111.	A/Kerala/RGCB_808/2015	03/02/2015	KU922545
112.	A/Delhi/RGCB_D10/2015	18/02/2015	MK080179
113.	A/Delhi/RGCB_D11/2015	18/02/2015	MK080180
114.	A/Delhi/RGCB_D16/2015	25/02/2015	MK080181
115.	A/Del/RGCB_D17/2015	28/02/2015	MK080182
116.	A/Del/RGCB_D26/2015	28/02/2015	MK080183
117.	A/Del/RGCB_D27/2015	28/02/2015	MK080184
118.	A/Del/RGCB_D28/2015	28/02/2015	MK080185
119.	A/Kerala/RGCB_2108/2017	03/03/2017	MF510838
120.	A/Kerala/RGCB_2158/2017	08/03/2017	MF510835
121.	A/Kerala/RGCB_2171/2017	09/03/2017	MF510836
122.	A/Kerala/RGCB_2247/2017	13/03/2017	MF510839
123.	A/Kerala/RGCB_2263/2017	14/03/2017	MF510840
124.	A/Kerala/RGCB_2302/2017	16/03/2017	MF510841
125.	A/Kerala/RGCB_2326/2017	21/03/2017	MF510837
126.	A/Montana/18/2009	04/05/2009	GQ377043
127.	A/Bayern/62/2009	29/04/2009	GQ365658
128.	A/Mexico/4108/2009	03/04/2009	GQ149651
129.	A/Arizona/01/2009	22/04/2009	GQ117067
130.	A/Korea/01/2009	02/05/2009	GQ131023
131.	A/Beijing/3/2009	20/05/2009	GQ225381
132.	A/Wisconsin/66/2009	16/09/2009	KC781310
133.	A/San Diego/INS03/2009	12/10/2009	CY055964
134.	A/India/GWL_DSC/2010	09/09/2009	JF293315
135.	A/Massachusetts/10/2013	03/06/2013	KF647947
136.	A/Iowa/19/2014	05/11/2014	KU592829
137.	A/Arizonal/15/2010	25/12/2014	KC881868
138.	A/California/80/2015	18/03/2015	KT836680
139.	A/Washington/19/2015	12/02/2015	KT836815
140.	A/Michigan/73/2015	14/12/2015	KX405963
141.	A/Arizona/48/2015	22/12/2015	KX406091
142.	A/Washington/07/2016	04/01/2016	KX919500
143.	A/Arizona/03/2016	01/01/2016	KX918452
144.	A/California/06/2016	01/01/2016	KY044980
145.	A/California/119/2016	16/06/2016	KY919284

146.	A/Arizona/01/2011	01/02/2011	KC881876
147.	A/St.Petersburg/100/2011	01/06/2011	CY121816
148.	A/Wisconsin/15/2011	11/02/2011	KC881725
149.	A/Michigan/45/2015	07/09/2015	MK622940

Supplementary Table S3. Details of HA segment of influenza A (H1N1) virus isolated during October 2009 to March 2017 from RGCB, Kerala along withother Indian and global representative isolates from NCBI influenza virus resource database used for constructing Maximum Likelihood phylogenetic tree represented in Supplementary Fig S6.

Supplementary figure S6

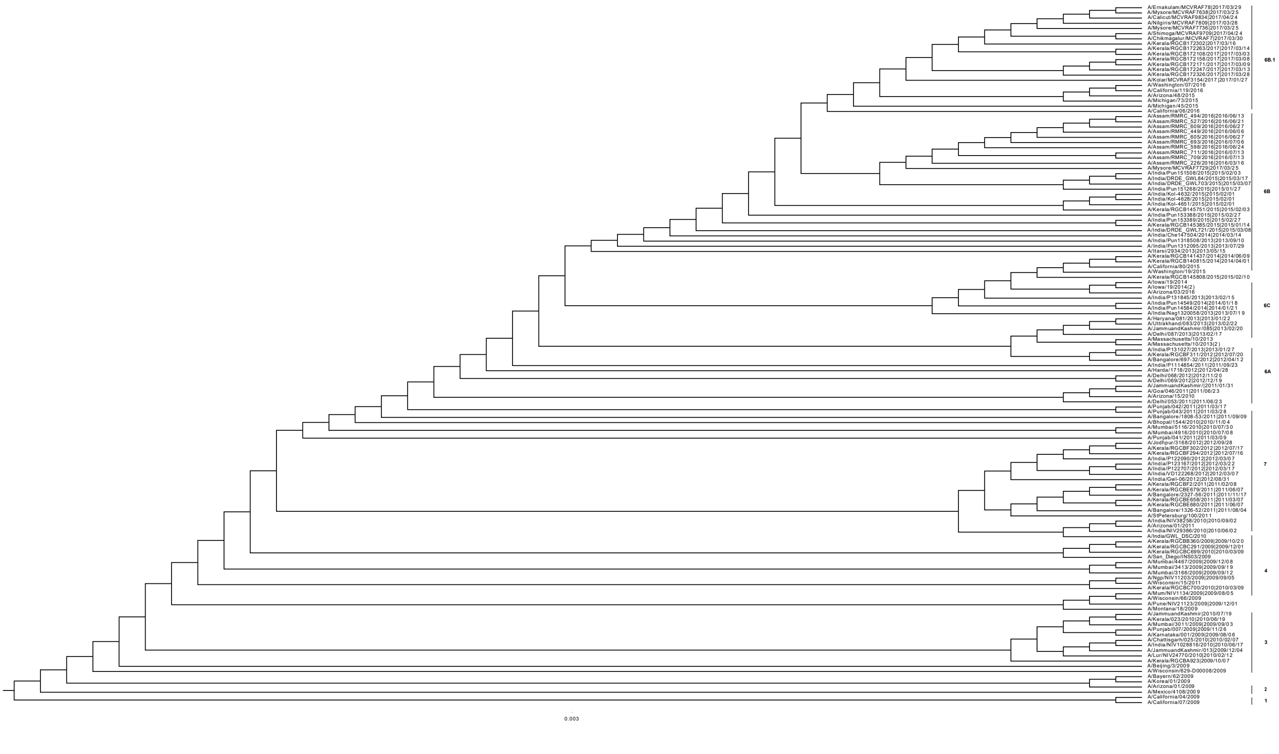

Supplementary Fig. S6. Maximum likelihood phylogeny of H1N1 strains isolated in India during 2009-2017 along with Vaccine strain and clade reference strains

Supplementary table S4

Sl.No.	Name of Isolate	Year of Isolation	Segment	Genebank ID
1.	A/Malappuram/MCVR2376/2009	14/12/2009	NA	KM985428
2.	A/Mumbai/183/2009	07/08/2009	NA	KJ958961
3.	A/Pune/NIV10278/2009	07/09/2009	NA	CY088640
4.	A/Bangalore/6206-12/2009	09/09/2009	NA	KR870128
5.	A/Delhi/NIV3610/2009	13/08/2009	NA	CY088675
6.	A/KOL/2449/2009	01/01/2009	NA	HM460505
7.	A/Mumbai/151/2009	07/08/2009	NA	KJ958945
8.	A/KOL/2465/2009	01/01/2009	NA	HM460506
9.	A/Mumbai/3051/2009	07/09/2009	NA	KJ958938
10.	A/Mumbai/3140/2009	09/09/2009	NA	KJ958977
11.	A/Ernakulam/MCVR5015/2010	21/07/2010	NA	KM985429
12.	A/Malappuram/MCVR3702/2010	2010/06/25	NA	KM985439
13.	A/Bangalore/6846-26/2010	23/08/2010	NA	KR870155
14.	A/Trivandrum/MCVR2613/2010	02/01/2010	NA	KM985432
15.	A/Bangalore/3934-28/2010	23/07/2010	NA	KR870141
16.	A/Bangalore/4163-30/2010	28/07/2010	NA	KR870144
17.	A/Bangalore/4976-17/2010	08/08/2010	NA	KR870146
18.	A/Bangalore/5279-29/2010	11/08/2010	NA	KR870149
19.	A/Bangalore/5532-05/2010	13/08/2010	NA	KR870151
20.	A/Bangalore/4161-22/2010	28/07/2010	NA	KR870143
21.	A/Trivandrum/MCVR7887/2011	20/08/2011	NA	KM985434
22.	A/India/GWL02/2011	24/08/2011	NA	JX262202
23.	A/Kottayam/MCVR6946/2011	20/06/2011	NA	KM985433
24.	A/India/P1114854/2011	23/09/2011	NA	KF280665
25.	A/India/GWL01/2011	24/08/2011	NA	JX262201
26.	A/Calicut/MCVR7353/2011	16/07/2011	NA	KU296288
27.	A/India/P1112874/2011	10/08/2011	NA	KF280657
28.	A/Kannur/MCVR8120/2011	06/09/2011	NA	KM985435
29.	A/Calicut/MCVR8470/2011	12/03/2011	NA	KM985441
30.	A/Udupi/MCVR7048/2011	28/06/2011	NA	KM985440
31.	A/Bangalore/2629-37/2012	04/09/2012	NA	KR870170
32.	A/Calicut/MCVR9427/2012	06/11/2012	NA	KM985431
33.	A/Bangalore/3432-40/2012	20/10/2012	NA	KR870173
34.	A/India/P121773/2012	24/02/2012	NA	KF280697
35.	A/India/P121778/2012	24/02/2012	NA	KF280705
36.	A/India/P121716/2012	24/02/2012	NA	KF280681
37.	A/Bangalore/2071-38/2012	02/08/2012	NA	KR870168
38.	A/Bangalore/2008-43/2012	30/07/2012	NA	KR870167
39.	A/India/P121939/2012	29/02/2012	NA	KF280713
40.	A/Assam/3774/2012	19/03/2012	NA	KU310640
41.	A/India/P132194/2013	18/02/2013	NA	KF280753
42.	A/India/P131845/2013	15/02/2013	NA	KF280745
43.	A/India/Nag132467/2013	31/01/2013	NA	KF280737
44.	A/India/P131027/2013	27/01/2013	NA	KF270584
45.	A/Calicut/MCVRAA1593/2013	06/05/2013	NA	KX907857
46.	A/India/Pun1415432/2014	20/10/2014	NA	KR052640

47.	A/India/Kol-T4/2015	01/02/2015	NA	KU695620
48.	A/India/Kol-4122/2015	13/03/2015	NA	KX789471
49.	A/Assam/5361/2015	05/06/2015	NA	KU310642
50.	A/India/Kol-S4481/2015	21/03/2015	NA	KX789426
51.	A/India/Pun151399/2015	01/02/2015	NA	KR052682
52.	A/India/Pun151192/2015	23/01/2015	NA	KR052654
53.	A/India/Kol-5025/2015	17/09/2015	NA	KX789475
54.	A/India/DRDE_GWL672/2015	05/03/2015	NA	KX078503
55.	A/India/Kol-3959/2015	09/03/2015	NA	KX789469
56.	A/India/Kol-4040/2015	11/03/2015	NA	KX789470
57.	A/Mexico/24057/2009	28/04/2009	NA	CY147925
58.	A/SouthCarolina/WRAIR1645P/2009	17/07/2009	NA	CY083282
59.	A/Singapore/ON816/2009	03/07/2009	NA	CY124133
60.	A/Russia/171/2009	12/11/2009	NA	CY054672
61.	A/Texas/45071344/2009	07/09/2009	NA	CY052809
62.	A/England/XFL00275/2009	05/05/2009	NA	CY065368
63.	A/Wisconsin/629-S0229/2009	01/01/2009	NA	CY051521
64.	A/Cambodia/NHRCC00009/2009	15/10/2009	NA	CY082986
65.	A/Jiangsu/S62/2009	10/11/2009	NA	HQ652615
66.	A/Korea/01/2009	02/05/2009	NA	GQ132185
67.	A/Singapore/GP3713/2010	03/08/2010	NA	JX309746
68.	A/Singapore/GP4406/2010	01/11/2010	NA	JX309912
69.	A/Uganda/MUWRP-222/2010	28/10/2010	NA	KJ690489
70.	A/Singapore/GP1430/2010	20/04/2010	NA	JX309331
71.	A/Singapore/GP185/2010	04/05/2010	NA	JX309397
72.	A/Thailand/SN08379/2010	04/06/2010	NA	KP637552
73.	A/NanPing/SWL1570/2010	26/12/2010	NA	KP019746
74.	A/Chile/64/2010	11/08/2010	NA	CY093167
75.	A/Thailand/CU-H1786/2010	20/03/2010	NA	CY080325
76.	A/NorthernIreland/05180459/2010	16/12/2010	NA	JX625952
77.	A/Beijing/HZ03/2011	14/01/2011	NA	JF316717
78.	A/Zhejiang/LS131/2011	01/01/2011	NA	KC524487
79.	A/SouthDakota/01/2011	12/01/2011	NA	KC881951
80.	A/Shanghai/3171T/2011	18/01/2011	NA	JN631042
81.	A/England/10740704/2011	09/01/2011	NA	LN846641
82.	A/Vermont/16/2011	22/12/2011	NA	KC891316
83.	A/NizhniiNovgorod/CRIE-BOU/2011	21/01/2011	NA	JN714541
84.	A/Iowa/04/2011	13/02/2011	NA	KC882083
85.	A/Santiago/p3d3/2011	18/08/2011	NA	KJ943384
86.	A/DistrictofColumbia/WRAIR0313/2011	28/01/2011	NA	CY090029
87.	A/NewYork/34/2012	17/04/2012	NA	KC891580
88.	A/SouthCarolina/3042/2012	03/10/2012	NA	CY130174
89.	A/VeraCruz/LACENRS-2842/2012	2012/08/23	NA	KY925741
90.	A/Tehran/7359/2012	17/12/2012	NA	KC842181
91.	A/Maine/06/2012	04/04/2012	NA	KC891122
92.	A/Montana/09/2012	26/03/2012	NA	KC891226
93.	A/SaoGabriel/LACENRS-1085/2012	10/07/2012	NA	KY925803
94.	A/Jordan/D3626/2012	13/12/2012	NA	KU933879
95.	A/Mexico/InDRE1474/2012	26/01/2012	NA	CY110744
96.	A/Ulaanbaatar/1687/2012	09/04/2012	NA	CY116605

97.	A/NanPing/SWL1640/2013	27/08/2013	NA	KP019794
98.	A/RosariodoSul/LACENRS-1832/2013	28/06/2013	NA	KY926246
99.	A/Helsinki/604/2013	12/02/2013	NA	KF559840
100.	A/Texas/10/2013	03/03/2013	NA	KF647970
101.	A/CzechRepublic/201/2013	30/01/2013	NA	KJ561764
102.	A/California/NHRC417074/2013	30/12/2013	NA	KJ635897
103.	A/Florida/61/2013	06/11/2013	NA	KT274398
104.	A/FuZhou/SWL1493/2013	27/05/2013	NA	KP019785
105.	A/NewHampshire/15/2013	18/12/2013	NA	KM409344
106.	A/NewYork/WC-LVD-13-014/2013	12/12/2013	NA	CY188923
107.	A/California/37/2014	04/10/2014	NA	KT836599
108.	A/California/10/2014	29/03/2014	NA	KM409028
109.	A/Fukuoka/DS4-117/2014	15/01/2014	NA	LC409143
110.	A/Florida/62/2014	28/10/2014	NA	KT880167
111.	A/Milano/88/2014	01/01/2014	NA	KU322059
112.	A/NewYork/WC-LVD-14-009/2014	19/01/2014	NA	CY189115
113.	A/Nagasaki/13N094/2014	13/03/2014	NA	LC033291
114.	A/Helsinki/219N/2014	27/01/2014	NA	KM219137
115.	A/Helsinki/219M/2014	27/01/2014	NA	KM219129
116.	A/Nagasaki/13N059/2014	29/01/2014	NA	LC033259
117.	A/NewHampshire/44/2015	04/12/2015	NA	KX004355
118.	A/Thailand/CU-B12418/2015	18/08/2015	NA	KX151309
119.	A/Idaho/39/2015	21/12/2015	NA	KX004685
120.	A/NewYork/72/2015	30/12/2015	NA	KX408213
121.	A/SaudiArabia/67/2015	10/12/2015	NA	MK228917
122.	A/SaudiArabia/58/2015	22/12/2015	NA	MK228911
123.	A/SaudiArabia/34/2015	26/10/2015	NA	MK228905
124.	A/Jeddah/KFAFH1568/2015	07/11/2015	NA	MF768629
125.	A/Iran/2/2015	02/11/2015	NA	KY288869
126.	A/NewHampshire/43/2015	03/12/2015	NA	KX004406
127.	A/NorthCarolina/19/2016	18/02/2016	NA	KX409301
128.	A/California/22/2016	22/01/2016	NA	KX006263
129.	A/Georgia/10/2016	01/01/2016	NA	KX408581
130.	A/Illinois/40/2016	01/04/2016	NA	KX915438
131.	A/California/193/2016	24/12/2016	NA	CY214284
132.	A/Tennessee/09/2016	29/02/2016	NA	KY044763
133.	A/California/34/2016	04/02/2016	NA	KX406677
134.	A/California/216/2016	13/12/2016	NA	CY220809
135.	A/Mississippi/5594/2016	31/01/2016	NA	CY210165
136.	A/Linkou/0077/2016	22/01/2016	NA	KY487383
137.	A/Louisiana/64/2017	14/11/2017	NA	MG830781
138.	A/Wisconsin/21/2017	23/01/2017	NA	CY220905
139.	A/Hawaii/25/2017	20/04/2017	NA	CY236261
140.	A/Mexico/8517/2017	18/01/2017	NA	MF593585
141.	A/Virginia/29/2017	22/04/2017	NA	CY236245
142.	A/Florida/102/2017	18/12/2017	NA	MG830714
143.	A/Texas/310/2017	05/12/2017	NA	CY263408
144.	A/SouthCarolina/03/2017	17/01/2017	NA	CY236068
145.	A/Arkansas/46/2017	21/12/2017	NA	MH083375
146.	A/Wyoming/30/2017	05/12/2017	NA	MH084250

Supplementary Table S4. Influenza A (H1N1) strains and gene segment from NCBI influenza virus resource database used in this study*.

*https://www.ncbi.nlm.nih.gov/genomes/FLU/Database/nph-select.cgi.

Supplementary figure S7

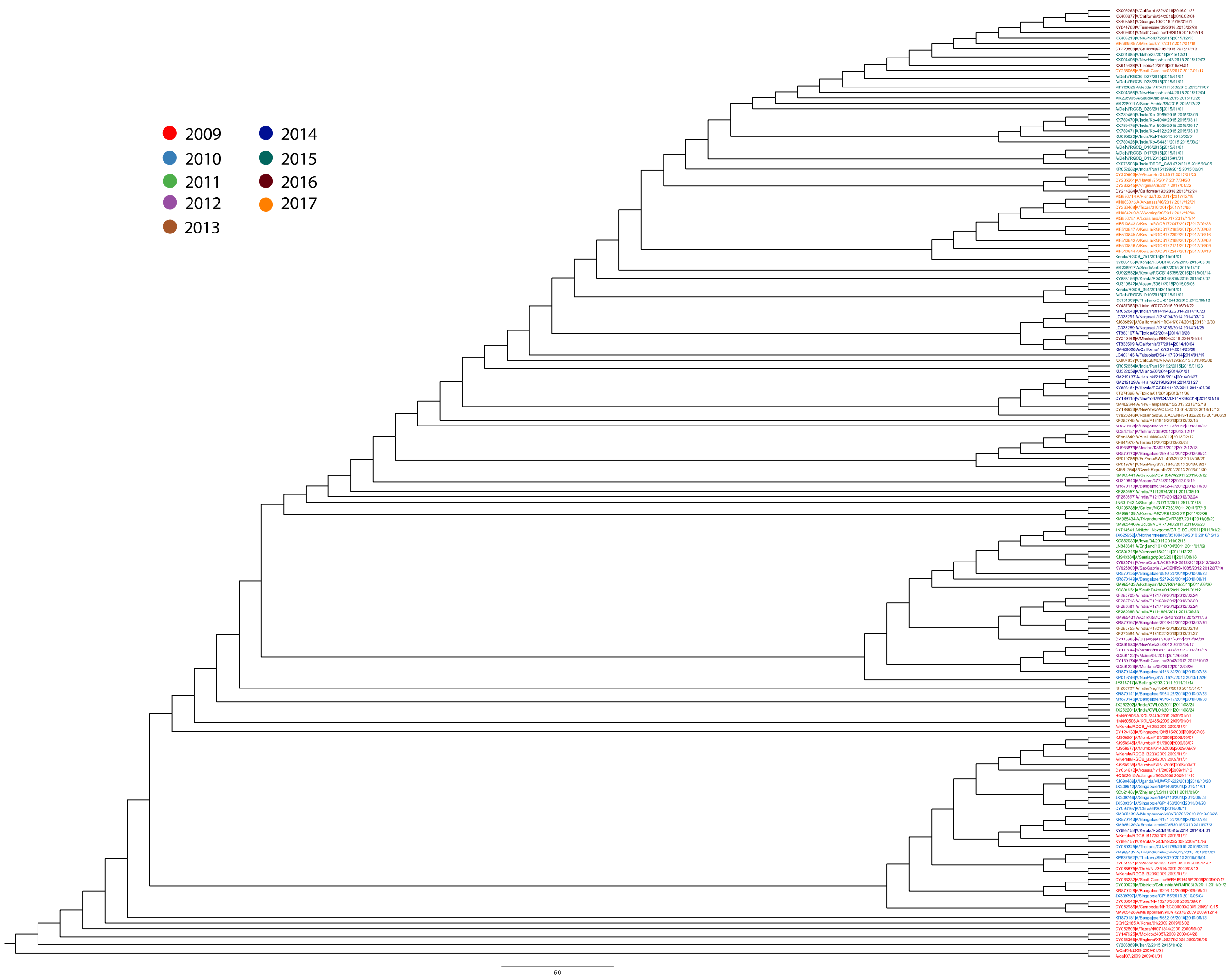

Supplementary Fig. S7. Maximum likelihood phylogeny of NA sequence of H1N1 strains circulating in India and globally during 2009-2017.

Supplementary figure S8

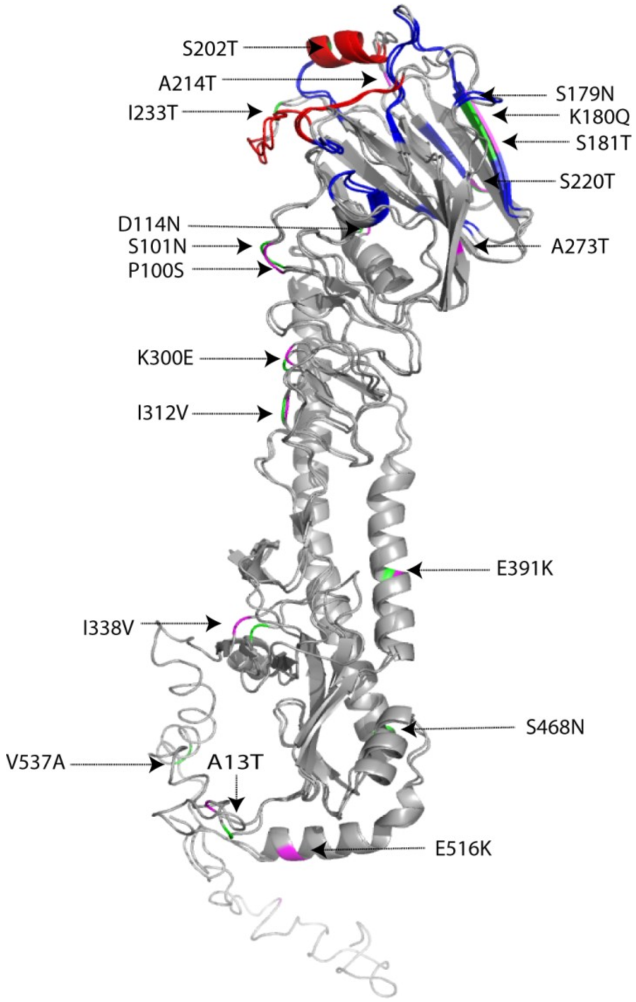

Supplementary Fig. S8. Superimposed structures of 2009 pandemic H1N1 HA, PDB: 3UBE and mutated HA**.** Binding site residues and the antigenic site residues are highlighted in red and blue, respectively. The mutated residues are highlighted in green in the mutated and purple in the 2009 pandemic H1N1 HA structure.

Supplementary table S5

YEAR 2009	YEAR 2010	YEAR 2011	YEAR 2012	YEAR 2013	YEAR 2014	YEAR 2015	YEAR 2016 *	YEAR 2017
								V13I
						I34V		I34V
						L40I		L40I
						N44S		N44S
**V106I**	V106I	V106I						
						N200S		N200S
		V241I	V241I	V241I		V241I		V241I
**N248D**	N248D	N248D	N248D		N248D			N248D
						V264I		V264I
						N270K		N270K
								D300N
								I314M
						I321V		I321V
		N369K	N369K	N369K		N369K		N369K
						N386K		N386K
						K432E		K432E

*Data of 2016 Indian strains not available.

Supplementary Table S5. Mutations observed in NA protein of Influenza A (H1N1) strains circulating in India between 2009 - 2017 with respect to reference strain (A/California/04/09) and vaccine strain (A/California/07/09). Only those mutations which are found universally or ≥ 90 percent of the sequence in the study are considered.

Supplementary table S6

Model	-lnL	K	BIC	delta	weight	cumWeight
**TPM1uf+G**	**8291.8662**	**350**	**19207.7549**	**0.0000**	**0.8663**	**0.8663**
**TIM1+G**	8290.1477	351	19211.8152	4.0602	0.1138	0.9801
**TVM+G**	8289.1698	352	19217.3565	9.6016	0.0071	0.9872
**TPM1uf+I+G**	8293.1497	351	19217.8192	10.0643	0.0057	0.9929
**HKY+G**	8301.2915	349	19219.1083	11.3534	0.0030	0.9959
**TPM3uf+G**	8297.7031	350	19219.4288	11.6739	0.0025	0.9984
**TIM1+I+G**	8291.6655	352	19222.3479	14.5929	0.0006	0.9990
**HKY+I+G**	8299.3793	350	19222.7811	15.0262	0.0005	0.9994
**TrN+G**	8299.7782	350	19223.5788	15.8239	0.0003	0.9998
**TPM2uf+G**	8301.2881	350	19226.5986	18.8437	0.0001	0.9998
**TVM+I+G**	8290.3308	353	19227.1757	19.4208	0.0001	0.9999
**GTR+G**	8290.8235	353	19228.1612	20.4063	0.0000	0.9999
**TPM3uf+I+G**	8298.6360	351	19228.7917	21.0367	0.0000	0.9999
**TrN+I+G**	8299.0808	351	19229.6813	21.9264	0.0000	1.0000
**TIM3+G**	8299.1223	351	19229.7642	22.0093	0.0000	1.0000
**TPM2uf+I+G**	8299.3783	351	19230.2764	22.5214	0.0000	1.0000
**TIM2+G**	8299.5340	351	19230.5877	22.8328	0.0000	1.0000
**GTR+I+G**	8288.8960	354	19231.8034	24.0484	0.0000	1.0000
**TIM3+I+G**	8297.2000	352	19233.4170	25.6621	0.0000	1.0000
**TIM2+I+G**	8297.9068	352	19234.8305	27.0756	0.0000	1.0000
**TPM1uf+I**	8307.3170	350	19238.6565	30.9016	0.0000	1.0000
**TIM1+I**	8306.0013	351	19243.5223	35.7674	0.0000	1.0000
**HKY+I**	8313.7807	349	19244.0867	36.3318	0.0000	1.0000
**TVM+I**	8304.4903	352	19247.9975	40.2425	0.0000	1.0000
**TrN+I**	8312.2407	350	19248.5039	40.7489	0.0000	1.0000
**TPM3uf+I**	8312.8050	350	19249.6325	41.8776	0.0000	1.0000
**TPM2uf+I**	8313.4425	350	19250.9076	43.1527	0.0000	1.0000
**GTR+I**	8303.2375	353	19252.9891	45.2342	0.0000	1.0000
**TIM3+I**	8311.5479	351	19254.6156	46.8606	0.0000	1.0000
**TIM2+I**	8312.2601	351	19256.0399	48.2850	0.0000	1.0000
**TVMef+G**	8363.9299	349	19344.3852	136.6303	0.0000	1.0000
**TPM1+G**	8374.8131	347	19351.1571	143.4021	0.0000	1.0000
**K80+G**	8379.7790	346	19353.5917	145.8367	0.0000	1.0000
**TrNef+G**	8376.1139	347	19353.7588	146.0038	0.0000	1.0000
**TPM3+G**	8377.1096	347	19355.7502	147.9952	0.0000	1.0000
**TPM2+G**	8377.5792	347	19356.6893	148.9343	0.0000	1.0000
**SYM+G**	8366.9737	350	19357.9698	150.2149	0.0000	1.0000
**TVMef+I+G**	8367.0939	350	19358.2104	150.4555	0.0000	1.0000
**TIM1ef+G**	8375.2497	348	19359.5276	151.7727	0.0000	1.0000
**TPM1+I+G**	8375.6122	348	19360.2526	152.4976	0.0000	1.0000
**TIM2ef+G**	8376.7496	348	19362.5273	154.7723	0.0000	1.0000
**K80+I+G**	8380.5813	347	19362.6934	154.9385	0.0000	1.0000
**SYM+I+G**	8365.6991	351	19362.9179	155.1629	0.0000	1.0000
**TIM3ef+G**	8377.5893	348	19364.2067	156.4518	0.0000	1.0000
**TIM1ef+I+G**	8374.0398	349	19364.6049	156.8500	0.0000	1.0000
**TPM3+I+G**	8377.8890	348	19364.8060	157.0511	0.0000	1.0000
**TPM2+I+G**	8378.2420	348	19365.5122	157.7572	0.0000	1.0000
**TrNef+I+G**	8379.0133	348	19367.0546	159.2997	0.0000	1.0000
**TIM3ef+I+G**	8376.3507	349	19369.2267	161.4717	0.0000	1.0000
**TIM2ef+I+G**	8376.5931	349	19369.7114	161.9565	0.0000	1.0000

**TVMef+I**	8381.5613	349	19379.6480	171.8931	0.0000	1.0000
**TPM1+I**	8389.1187	347	19379.7683	172.0133	0.0000	1.0000
**TPM3+I**	8391.5711	347	19384.6732	176.9183	0.0000	1.0000
**SYM+I**	8380.5235	350	19385.0695	177.3146	0.0000	1.0000
**TIM1ef+I**	8388.1093	348	19385.2466	177.4917	0.0000	1.0000
**K80+I**	8395.6565	346	19385.3467	177.5918	0.0000	1.0000
**TPM2+I**	8392.2563	347	19386.0436	178.2886	0.0000	1.0000
**TrNef+I**	8392.9889	347	19387.5087	179.7538	0.0000	1.0000
**TIM3ef+I**	8392.0245	348	19393.0771	185.3222	0.0000	1.0000
**TIM2ef+I**	8392.6219	348	19394.2720	186.5170	0.0000	1.0000
**TPM1uf**	8439.0697	349	19494.6646	286.9097	0.0000	1.0000
**TrN**	8440.0819	349	19496.6890	288.9341	0.0000	1.0000
**TIM1**	8437.0699	350	19498.1623	290.4074	0.0000	1.0000
**HKY**	8445.4405	348	19499.9091	292.1541	0.0000	1.0000
**TVM**	8436.3931	351	19504.3060	296.5510	0.0000	1.0000
**TPM3uf**	8444.8025	349	19506.1303	298.3754	0.0000	1.0000
**TPM2uf**	8445.4184	349	19507.3622	299.6073	0.0000	1.0000
**GTR**	8434.4198	352	19507.8565	300.1016	0.0000	1.0000
**TIM3**	8442.8245	350	19509.6715	301.9165	0.0000	1.0000
**TIM2**	8443.4267	350	19510.8759	303.1210	0.0000	1.0000
**TVMef**	8519.0074	348	19647.0429	439.2879	0.0000	1.0000
**TPM1**	8526.5862	346	19647.2061	439.4512	0.0000	1.0000
**TrNef**	8526.8800	346	19647.7937	440.0388	0.0000	1.0000
**K80**	8531.6040	345	19649.7445	441.9896	0.0000	1.0000
**TPM3**	8529.5188	346	19653.0712	445.3163	0.0000	1.0000
**TIM1ef**	8526.1849	347	19653.9007	446.1457	0.0000	1.0000
**TPM2**	8530.7530	346	19655.5397	447.7848	0.0000	1.0000
**SYM**	8520.2014	349	19656.9280	449.1731	0.0000	1.0000
**TIM3ef**	8529.1171	347	19659.7651	452.0102	0.0000	1.0000
**TIM2ef**	8530.3519	347	19662.2348	454.4799	0.0000	1.0000
**F81+G**	8671.5454	348	19952.1188	744.3639	0.0000	1.0000
**F81+I+G**	8670.1999	349	19956.9252	749.1703	0.0000	1.0000
**F81+I**	8686.6846	348	19982.3972	774.6423	0.0000	1.0000
**JC+G**	8754.5662	345	20095.6690	887.9140	0.0000	1.0000
**JC+I+G**	8755.4681	346	20104.9699	897.2150	0.0000	1.0000
**JC+I**	8768.8389	345	20124.2142	916.4593	0.0000	1.0000
**F81**	8819.1534	347	20239.8376	1032.0827	0.0000	1.0000
**JC**	8905.8088	344	20390.6568	1182.9019	0.0000	1.0000

Supplementary Table S6. JmodelTest statistics performed for H1N1 HA data - Best model according to the Bayesian Information criteria (BIC) is highlighted in Bold.
